# Obstacle Avoidance Path Planning for Robotic Arms Using a Multi-Strategy Collaborative Bidirectional RRT* Algorithm

**DOI:** 10.3390/s26041376

**Published:** 2026-02-22

**Authors:** Xiangchen Ku, Erzhou Zhu, Sen Li

**Affiliations:** School of Mechatronics Engineering, Henan University of Science and Technology, Luoyang 471003, China; 230320010091@stu.haust.edu.cn (E.Z.); 230320010069@stu.haust.edu.cn (S.L.)

**Keywords:** robotic arm, path planning, dynamic ellipsoidal sampling strategy, dynamic goal-biased strategy, improved RRT* algorithm

## Abstract

In response to issues such as insufficient bias in random sampling, low convergence efficiency, inadequate path search efficiency, and lack of path smoothness encountered by the traditional RRT* algorithm during path planning, an improved algorithm is proposed. First, a dynamic ellipsoidal sampling strategy is introduced, which accelerates the exploration of the path space by adaptively adjusting the sampling region. Additionally, a bidirectional RRT* algorithm is employed, establishing two alternately growing search trees to perform bidirectional search, thereby effectively enhancing the convergence speed of the algorithm. Second, a dynamic goal-biased strategy is adopted, which greedily guides the random tree to grow rapidly toward the goal point, thereby improving planning efficiency. A heuristic search scheme is integrated with the RRT* algorithm to further increase convergence speed. A random sampling expansion strategy is utilized to guide the tree to expand into unexplored regions, avoiding local minima while ensuring global search capability. Local reconnection optimization is applied to reduce the cumulative path cost of new nodes while balancing path length, smoothness, and safety. To reduce the number of iterations, an improved artificial potential field method is incorporated into the growth process of the bidirectional random search trees, providing directional guidance for their expansion. Finally, path pruning techniques are applied to eliminate redundant nodes from the initial path, and a cubic B-spline interpolation algorithm is used to smooth the pruned path, generating a final trajectory with continuous curvature suitable for tracking. Quantitative analysis of simulation experiments in three-dimensional space shows that in both simple and complex environments, compared with the RRT, GB-RRT, BI-RRT, APF-RRT, and BI-APF-RRT* algorithms, the improved RRT* algorithm reduces planning time by approximately 58–90%, decreases the number of path nodes by about 31–91%, and shortens path length by around 8–20%, demonstrating the superiority of the proposed algorithm.

## 1. Introduction

The continuous advancement of computers and intelligent technologies is driving robotic applications toward greater intelligence and diversification while simultaneously imposing higher demands on their overall performance [1,2]. The core task of robotic arm path planning is to search for a feasible trajectory that safely moves the arm from its initial configuration to a target configuration without collisions under kinematic constraints and obstacle avoidance requirements. To enable efficient motion in complex environments and precise task execution at the target location, advanced path planning algorithms are essential for generating viable trajectories. Consequently, path planning algorithms constitute a critical component in the development of robotic arm technologies [3,4].

Current path planning algorithms can be broadly categorized into (1) graph-based search methods, such as Dijkstra’s algorithm [5], A* [6], and D* [7]; (2) sampling-based methods, including the Rapidly exploring Random Tree (RRT) and Probabilistic Roadmap (PRM); and (3) bio-inspired intelligent algorithms, such as genetic algorithms, ant colony optimization, particle swarm optimization, and neural network–based approaches—particularly those integrated with deep learning. Among these, the RRT algorithm stands out as a classical representative of sampling-based planners. Owing to its efficient exploration capability and strong adaptability to complex environments, RRT has gained widespread application and recognition in engineering practice [8]. The RRT algorithm was first proposed by LaValle et al. in 1998 as a tree-structured, randomized planning method designed to efficiently address path planning problems in high-dimensional spaces. However, its inherent randomness leads to drawbacks such as long computation time and low convergence efficiency. To address these limitations, Wang et al. introduced a goal-biased sampling strategy. This approach uses a bias threshold compared against a random number to guide the random tree toward the goal direction with a certain probability, thereby reducing redundant nodes and improving search efficiency. Building upon basic RRT, Karaman et al. proposed the RRT* algorithm, which incorporates a rewiring mechanism. After inserting a new node, RRT* re-optimizes connections within its neighborhood by re-selecting parent nodes to progressively reduce path cost, ultimately achieving asymptotic optimality. Gammell et al. further advanced this with Informed RRT*, which introduces an ellipsoidal informed sampling strategy: once an initial feasible path is found, subsequent sampling is confined to an ellipse with the start and goal as foci, significantly accelerating convergence toward the optimal solution. Subsequent research has further extended RRT* for diverse scenarios. Qi et al. developed a Multi-Objective Dynamic RRT* (MOD-RRT*) for navigation in unknown dynamic environments [9]. Qureshi et al. [10] proposed P-RRT*, which integrates artificial potential fields into the RRT* framework, using attractive forces toward the goal to guide tree growth and enhance global search efficiency. GUO et al. [11] proposed the DBVSB-P-RRT* algorithm, which incorporates an adaptive deflection sampling function, an adaptive attraction function based on the artificial potential field (APF), a variable-step expansion heuristic strategy, and a novel collision detection heuristic method. This algorithm possesses rapid planning capabilities, making it particularly suitable for path generation in emergency scenarios for mobile robots, enabling efficient output of trajectories that satisfy constraint conditions. However, this method offers limited improvement in path quality and is currently confined to two-dimensional path planning problems, without yet addressing relevant factors in multidimensional space planning. Zhen et al. [12] proposed the HS-APF-RRT* algorithm, which integrates a hierarchical terrain sampling mechanism, a slope-aware artificial potential field, and a dynamic 8/16-neighborhood expansion strategy. This algorithm can plan the shortest and highest-quality paths in complex environments. It not only achieves initial feasible solutions faster and significantly reduces the number of iterations and energy consumption but also completely avoids hazardous areas, providing an efficient, safe, and robust solution for path planning in complex environments. Jeong et al. introduced Q-RRT*, which expands the parent selection range to include grandparent nodes during rewiring, significantly improving both search efficiency and path quality [13]. Li et al. combined P-RRT* and Q-RRT* into PQ-RRT*, leveraging their complementary strengths to accelerate convergence and enhance planning efficiency [14]. Wei et al. proposed Smooth RRT (S-RRT), which generates kinematically feasible and smooth trajectories through a maximum curvature-constrained optimization strategy; however, its reliance solely on goal bias during tree expansion limits its search efficiency and results in substantial deviation between the optimized and initial paths [15]. Nasir et al. presented RRT*-Smart, which improves efficiency through path optimization and intelligent sampling, but it was validated only in 2D environments and still produces paths with redundant turns. Liao et al. [16] proposed F-RRT*, which actively assigns the optimal parent node to each new sample to reduce cumulative path cost, yet its parent selection strategy exhibits limited adaptability in complex environments, leading to slow convergence. Cong et al. [17] introduced FF-RRT*, which fuses F-RRT* with Fast-RRT* and refines the hybrid sampling strategy, effectively overcoming limitations of prior methods and reducing convergence time—though sampling efficiency remains suboptimal in obstacle-dense scenarios. Chai et al. [18] proposed RJ-RRT, which employs a greedy sampling space reduction strategy to minimize redundant tree expansion; however, it may still suffer from slow convergence in environments with uneven obstacle distributions. Ganesan et al. [19] developed a hybrid sampling-based RRT* variant that combines uniform and non-uniform sampling with dynamic sampling domain adjustment, effectively balancing exploration and exploitation to enhance planning efficiency and adaptability in complex environments. Qie et al. [20] introduced Target-biased Fast RRT* (TF-RRT*), which biases samples toward the goal to accelerate convergence. Ji et al. [21] proposed E-RRT*, which replaces straight-line connections with elliptical arcs to speed up convergence and incorporates HRM angle constraints to produce more kinematically compliant paths with reduced redundancy. Ding et al. [22] presented EP-RRT, which integrates bidirectional search with dynamic region contraction: it first rapidly generates an initial path using an RRT-Connect-like dual-tree mechanism, then applies heuristic sampling within a shrinking region to iteratively prune redundant nodes and refine the path toward optimality. Chen et al. proposed a bidirectional RRT* variant that decouples path expansion from optimization [23]. Chao et al. combined RRT* with grid-based search to create GB-RRT*, which enhances performance in narrow passages and complex obstacle fields without requiring a predefined roadmap [24]. Liu Xiaosong et al. improved P-RRT* by refining the potential field guidance and introducing a dynamic step-size adjustment mechanism, significantly accelerating convergence. Cheng et al. [25] proposed an RRT-Connect-based improvement featuring adaptive step size and four concurrent trees rooted at the start, goal, and two fixed sampling points, greatly enhancing spatial coverage and convergence speed. Song Junhui et al. integrated an Adaptive RRT* based on an Improved Potential function (AIP-RRT*) with a Dynamic Gravitational Field–Artificial Potential Field (DGF-APF) method, synergistically combining sampling guidance and gradient descent to overcome the local minima problem of APF and the randomness of RRT. Huang et al. [26] proposed Agile-RRT*, which improves convergence via adaptive goal bias but is limited to 2D scenarios and lacks generalization to 3D or higher-dimensional spaces.

Based on existing research, this paper proposes an enhanced RRT* algorithm to address the limitations of the RRT* algorithm in terms of sampling guidance, search efficiency, and convergence speed. First, a dynamic ellipsoidal sampling strategy is introduced to adaptively shrink the sampling region, accelerating spatial exploration. Simultaneously, a bidirectional RRT* framework is adopted, enhancing convergence efficiency through alternating expansion from both the start and goal points. Second, a dynamic goal-biasing strategy is combined with a heuristic search mechanism to strengthen goal orientation while maintaining global exploration capability. The improved artificial potential field method is employed to guide the tree expansion direction, further reducing the number of iterations. The algorithm also integrates local rewiring optimization, which simultaneously optimizes path cost during node expansion, balancing path length, smoothness, and safety. Finally, path pruning technology is applied to remove redundant nodes, and the path is smoothed using a cubic B-spline interpolation algorithm, generating a final trajectory with continuous curvature suitable for execution.

## 2. Robotic Arm Kinematic Modeling and Collision Detection

### 2.1. Robotic Arm Kinematic Modeling

**Figure 1:**
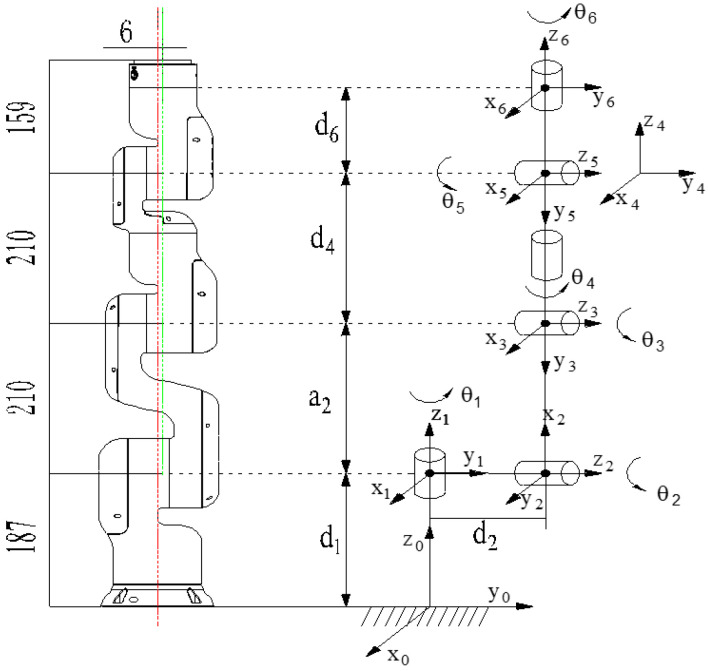
Robotic arm structure. The letters on the black arrows represent the distance between the connecting rods and the length of the connecting rods, while the letters on the curved arrows represent the angle of rotation of the connecting rods.

The experimental platform used in this study is the JAKA six-degrees-of-freedom (6-DOF) robotic arm, which consists of six revolute joints. The kinematic model of the arm is established using the standard Denavit–Hartenberg (D-H) parameter method. Figure 1 illustrates the physical configuration of the robotic arm, and the corresponding D-H parameters are listed in Table 1. 

The geometric relationship between each pair of adjacent links in a robot can be fully and precisely described mathematically by four parameters: θ, d, a, and α, where θ is the joint angle (the link twist angle), d is the link offset (the distance along the previous joint axis), a is the link length (the distance along the common normal), and α is the link twist (the angle between two adjacent joint axes about the common normal). The homogeneous transformation matrix Tii−1 is computed as shown in Equation (1), which is obtained by left-multiplying four elementary transformation matrices in sequence, specifically expressed as

(1) Tii−1=R(x,αi−1)×T(x,ai−1)×R(z,θi)×T(z,di)

In the equation, $R(x,\alpha_{i-1})$ denotes a rotation about the $x_{i-1}$-axis by angle $\alpha_{i-1}$, $T(x,a_{i-1})$ denotes a translation along the $x_{i-1}$-axis by distance $a_{i-1}$, $R(z,\theta_{i})$ denotes a rotation about the $z_{i}$-axis by angle $\theta_{i}$, and $T(z,d_{i})$ denotes a translation along the $z_{i}$-axis by distance $d_{i}$.

By expanding and deriving Equation (1), the general expression of the homogeneous transformation matrix can be obtained, which is specifically given as follows:

(2) Tii−1=cθi−sθi0ai−1sθicαi−1cθicαi−1−sαi−1−disθi−1sθisαi−1cθisαi−1cαi−1dicαi−10001

In the equation, $c\theta_{i}=\cos(\theta_{i})$, and $s\theta_{i}=\sin(\theta_{i})$.

Based on the above equations, the homogeneous transformation matrix T60 from the base coordinate frame to the end-effector coordinate frame of the robotic arm can be obtained, and its expression is as follows:

(3) T60=T01(θ1)T12(θ2)⋯T56(θ6)

### 2.2. Collision Detection

**Figure 2:**
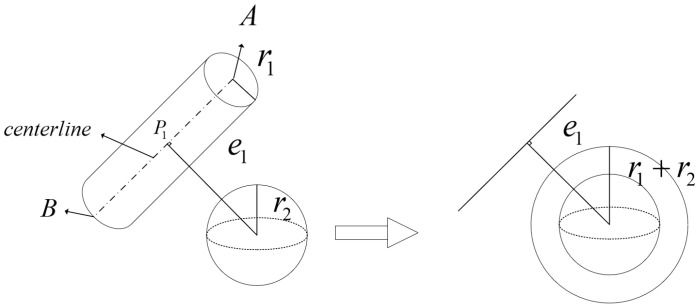
Collision detection model. The black dotted line represents the axis of the cylinder, and the black dashed line represents the centerline of the sphere.

To achieve high-precision collision detection and efficient obstacle-avoidance path planning, this paper employs the cylindrical enveloping method to construct a collision model for the robotic arm. To simplify the analysis, obstacles are approximated using minimum bounding spheres. Consequently, the collision detection problem between the robotic arm and obstacles is transformed into a calculation of the distance between cylinders (representing arm links) and spheres (representing obstacles). By merging the radius $r_{1}$ of the cylindrical envelope of the arm link with the radius $r_{2}$ of the obstacle’s bounding sphere, the collision detection problem is further simplified to determining whether a sphere intersects with a line segment (representing the central axis of the cylinder). Figure 2 visually illustrates the geometric model used for collision detection in this work.

Let the center of the obstacle’s bounding sphere be $O^{\prime }$ with a radius $r_{2}$. Draw a perpendicular line from the sphere center to the central axis of the robotic arm link, and denote the foot of the perpendicular as $P_{1}(x_{P_{1}},y_{P_{1}},z_{P_{1}})$, with the distance $e_{1}$. Let the endpoints of the link’s central axis be A and B, with coordinates $\left(x_{1},y_{1},z_{1}\right)$ and $\left(x_{2},y_{2},z_{2}\right)$, respectively. The direction vector of the link’s central axis is then $\left(x_{2}-x_{1},y_{2}-y_{1},z_{2}-z_{1}\right)$. Thus, the parametric (point-direction) form of the spatial line representing the link’s central axis can be expressed as

(4) $$\frac{x-x_{1}}{x_{2}-x_{1}}=\frac{y-y_{1}}{y_{2}-y_{1}}=\frac{z-z_{1}}{z_{2}-z_{1}}=t_{2}\in (0,1)$$

where $t_{2}$ is a scalar parameter.

From the orthogonality condition, it follows that

(5) $$(x_{2}-x_{1})(x_{O^{\prime }}-x_{P_{1}})+(y_{2}-y_{1})(y_{O^{\prime }}-y_{P_{1}})+(z_{2}-z_{1})(z_{O^{\prime }}-z_{P_{1}})=0$$

Substituting Equation (4) into Equation (5) yields the coordinates of point $P_{1}$. The collision status between the robotic arm and the obstacle can then be determined by comparing the distance $e_{1}$ with the combined radius $r_{1}+r_{2}$. Specifically, if $e_{1}>r_{1}+r_{2}$, no collision occurs; otherwise, a collision is detected.

## 3. Related Work

### 3.1. RRT* Algorithm

**Figure 3:**
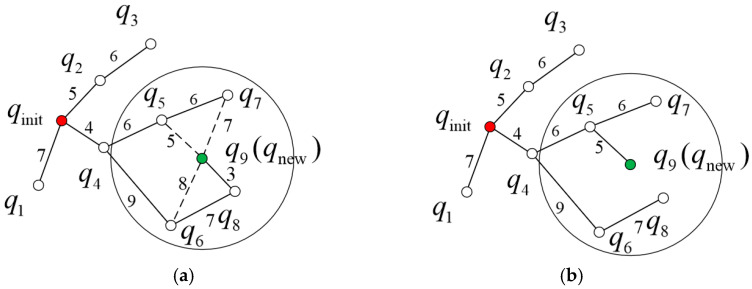
Reconnect to the parent node. (**a**) Finding the parent node. (**b**) Selecting the parent node. The red dot represents the starting point, the green dot represents a new node, and the dashed line represents the candidate parent nodes before reconnecting to the parent node.

The RRT* algorithm enhances the basic RRT framework by incorporating a real-time optimization mechanism. During the expansion of the random search tree, it simultaneously optimizes the path by evaluating the path costs of multiple neighboring nodes near the new sample and dynamically selecting a globally superior connection. This algorithm primarily features two key improvements, the first being the rewiring (parent reconnection) strategy. The parent reconnection process is illustrated in Figure 3. In Figure 3a, $q_{\mathrm{init}}$ denotes the start point, and $q_{i}(i=1,2,\cdots ,9)$ represents the node. The numbers labeled on the lines connecting the nodes indicate the Euclidean distances between the corresponding node pairs. A circle is drawn centered at $q_{9}(q_{\mathrm{new}})$ with radius r, yielding potential parent candidates within the circle: $q_{5}$, $q_{6}$, $q_{7}$, and $q_{8}$. The original path is $q_{\mathrm{init}}\to q_{4}\to q_{6}\to q_{8}\to q_{9}$, with a total path cost of 23. When $q_{5}$, $q_{6}$, and $q_{7}$ are considered as parent nodes, the corresponding paths are $q_{\mathrm{init}}\to q_{4}\to q_{5}\to q_{9}$, $q_{\mathrm{init}}\to q_{4}\to q_{6}\to q_{9}$, and $q_{\mathrm{init}}\to q_{4}\to q_{5}\to q_{7}\to q_{9}$, with path costs of 15, 21, and 23, respectively. After comparison, the path with $q_{5}$ as the parent node yields the lowest cost. Therefore, the parent of $q_{9}$ is updated from $q_{8}$ to $q_{5}$. The resulting updated path is shown in Figure 3b.

**Figure 4:**
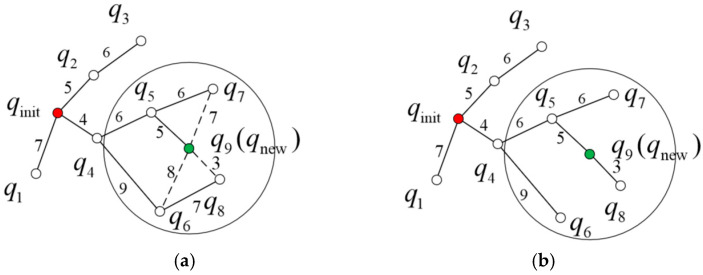
Rewiring. (**a**) Parent node change. (**b**) Rewiring. The red dot represents the starting point, the green dot represents a new node, and the dashed line indicates the changes to the parent node before rewiring.

The detailed rewiring process is illustrated in Figure 4. In Figure 4a, the nodes inside the circle are $q_{6}$, $q_{7}$, and $q_{8}$, whose current parent nodes are $q_{4}$, $q_{5}$, and $q_{6}$, respectively. The corresponding paths are $q_{\mathrm{init}}\to q_{4}\to q_{6}$, $q_{\mathrm{init}}\to q_{4}\to q_{5}\to q_{7}$, and $q_{\mathrm{init}}\to q_{4}\to q_{6}\to q_{8}$, with path costs of 13, 16, and 20, respectively. When considering $q_{9}(q_{\mathrm{new}})$ as a potential new parent for $q_{6}$, $q_{7}$, and $q_{8}$, the resulting paths become $q_{\mathrm{init}}\to q_{4}\to q_{5}\to q_{9}\to q_{6}$, $q_{\mathrm{init}}\to q_{4}\to q_{5}\to q_{9}\to q_{7}$, and $q_{\mathrm{init}}\to q_{4}\to q_{5}\to q_{9}\to q_{8}$, with updated path costs of 23, 22, and 18, respectively. After comparison, the path costs when $q_{9}(q_{\mathrm{new}})$ serves as the parent of $q_{6}$ and $q_{7}$ (i.e., 23 and 22) are higher than their original costs (13 and 16); therefore, their parent nodes remain unchanged as $q_{4}$ and $q_{5}$, respectively. However, when $q_{9}(q_{\mathrm{new}})$ is set as the parent of $q_{8}$, the new path cost (18) is lower than the original cost (20). Hence, the parent of $q_{8}$ is updated from $q_{6}$ to $q_{9}(q_{\mathrm{new}})$. The resulting updated tree structure is shown in Figure 4b.

### 3.2. BI-APF-RRT* Algorithm

The APF-RRT* algorithm is built upon the RRT* framework and effectively integrates the artificial potential field (APF) method. In this approach, the goal exerts an attractive force while obstacles generate repulsive forces, and random sample points are also assigned a certain degree of attraction toward the goal. During path expansion, instead of using the original random extension strategy of RRT*, the algorithm determines the direction for generating new nodes based on the resultant vector of attraction and repulsion forces. This enhancement significantly improves the random tree’s goal-directed exploration capability and convergence speed, enabling more efficient generation of feasible paths in complex environments.

**Figure 5:**
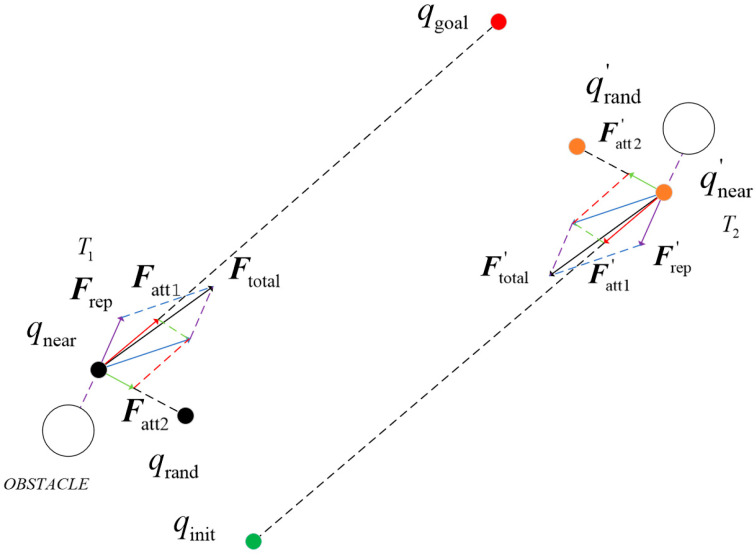
Schematic diagram of force analysis on $q_{\mathrm{near}}$ in the BI-APF-RRT* algorithm. The purple arrow represents the direction of repulsive force, the red and green arrows represent the direction of attractive force, and the black arrow represents the direction of the resultant force.

The BI-APF-RRT* algorithm improves upon the APF-RRT* algorithm by extending its search structure from a unidirectional random tree to a bidirectional random tree, thereby significantly enhancing the overall search efficiency. In the BI-APF-RRT* algorithm, the expansion procedure for generating a new sample node $q_{\mathrm{new}}$ in both random search trees is identical to that in the APF-RRT *algorithm. The attractive forces $F_{\mathrm{att}1}$ and $F_{\mathrm{att}2}$ are exerted on $q_{\mathrm{near}}$ by $q_{\mathrm{rand}}$ and $q_{\mathrm{goal}}$, respectively, while the repulsive force $F_{\mathrm{rep}}$ is generated by obstacles acting on $q_{\mathrm{near}}$. The growth direction of $q_{\mathrm{new}}$ is determined by the resultant force vector of these three components. Figure 5 illustrates the force analysis diagram for the BI-APF-RRT* algorithm. In Figure 5, $q_{\mathrm{rand}}$ is a random sample point; $q_{\mathrm{init}}$ and $q_{\mathrm{goal}}$ denote the start and goal points, respectively; and $q_{\mathrm{new}}$ represents a newly generated node. $T_{1}$ and $T_{2}$ are the two random search trees. Circular markers in the figure represent obstacles, $F_{\mathrm{att}1}$ and ${F_{\mathrm{att}1}}^{\prime }$, denote the attractive forces exerted on $q_{\mathrm{near}}$ by the goal point $q_{\mathrm{goal}}$ and the start point $q_{\mathrm{init}}$, respectively. $F_{\mathrm{att}2}$ and ${F_{\mathrm{att}2}}^{\prime }$ represent the attractive forces exerted on $q_{\mathrm{near}}$ by $q_{\mathrm{rand}}$ and ${q_{\mathrm{rand}}}^{\prime }$, respectively (corresponding to the respective tree expansions). $F_{\mathrm{rep}}$ and ${F_{\mathrm{rep}}}^{\prime }$ denote the repulsive forces exerted by obstacles on $q_{\mathrm{near}}$ and ${q_{\mathrm{near}}}^{\prime }$ for the two trees. The resultant forces, $F_{\mathrm{total}}$ and ${F_{\mathrm{total}}}^{\prime }$, are obtained by vectorially summing the corresponding three forces using the parallelogram law. The new node of the start tree is extended along the direction of $F_{\mathrm{total}}$, while the new node of the goal tree is extended along the direction of ${F_{\mathrm{total}}}^{\prime }$.

## 4. Improved RRT* Algorithm

### 4.1. Dynamic Ellipsoidal Sampling Strategy

To address the issues of uneven distribution and low-quality samples in the traditional RRT algorithm’s random sampling strategy, this paper introduces a dynamic ellipsoidal sampling mechanism. This strategy adaptively adjusts the size and scope of the sampling ellipsoid based on the real-time progress of path search and environmental complexity, thereby enhancing the relevance and effectiveness of the sampled points.

#### 4.1.1. Ellipsoidal Sampling Parameters

**Figure 6:**
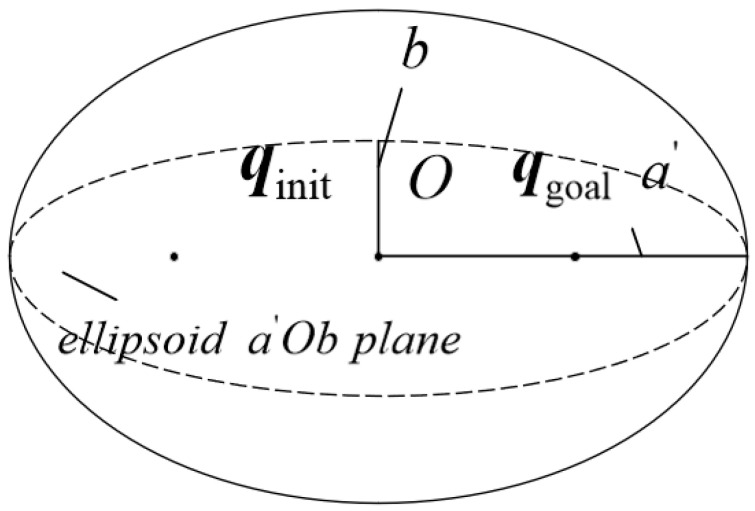
Ellipsoid (3D). The black dots represent the foci and circular points of the ellipse, the solid black lines represent the semi-major and semi-minor axes of the ellipse, and the black dashed lines represent the centerline of the ellipse.

**Figure 7:**
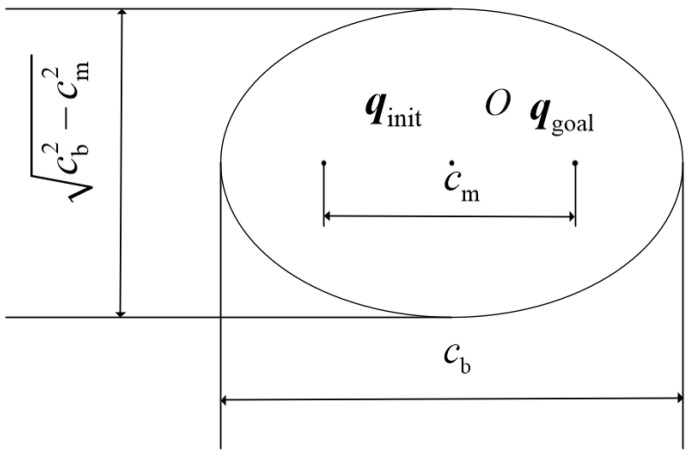
Ellipsoid $a^{\prime }Ob$ plane. The black dots represent the foci of the ellipse and the ellipse points, and the black solid lines represent the semi-major axis and semi-minor axis of the ellipse.

The 3D shape of the ellipsoid and its parametric representation in the plane are shown in Figure 6 and Figure 7, respectively.

(6) $$a^{\prime }=\frac{c_{b}}{2},b=\frac{\sqrt{c_{b}^{2}-c_{m}^{2}}}{2}$$

where $q_{\mathrm{init}}$ and $q_{\mathrm{goal}}$ are the foci of the ellipsoid, $c_{m}$ is the distance between the two foci, $c_{b}$ is the length of the major axis of the ellipsoid, and O is the center of the ellipsoid (i.e., the midpoint of the line segment joining the two foci).

#### 4.1.2. Dynamic Adjustment Formula for Ellipsoid Parameters

The adjustment formula for the semi-major axis $a^{\prime }$ is

(7) $$a_{k}^{\prime }=a_{0}^{\prime }\cdot {\left(1+\frac{1}{n}\right)}^{k}$$

The adjustment formula for the semi-minor axis b is

(8) $$b_{k}=b_{0}\cdot {\left(1+\frac{1}{n}\right)}^{k}$$

In the equation, $a^{\prime }$ is the semi-major axis of the ellipsoid, b is the semi-minor axis of the ellipsoid, n is the environmental complexity factor (related to obstacle penetrability), and k is the iteration count. The adjustment period is defined as updating the parameters once every iter_per_expansion iterations (e.g., if iter_per_expansion = 100, parameters are updated after every 100 iterations). iter_per_expansion indicates how many iterations occur before updating the ellipsoid parameters; $b_{0}$ is the initial value of b after each update cycle (determined by environmental complexity, with a new $b_{0}$ value defined after each update based on current environmental complexity); $a_{0}^{\prime }$ is the initial value of the semi-major axis $a^{\prime }$ at the beginning of each iteration cycle.

The environmental complexity factor n is defined as

(9) $$\begin{array}{l} n=n_{b}+\lambda_{1}C_{v}+\lambda_{2}C_{n}+\lambda_{3}C_{h}\\ C_{v}=\frac{V_{obs,local}}{V_{local}}\\ C_{n}=\tanh(\frac{m_{local}}{m_{ref}})\\ C_{h}=\frac{N_{fail,recent}}{N_{total,recent}} \end{array}$$

In the formula, $n_{b}$ is the base complexity, set as $n_{b}=8$; $C_{v}$ represents the volumetric complexity, where $V_{obs,local}$ is the total volume of obstacles within the local space, and $V_{local}$ is the volume of the local sphere; $C_{n}$ represents the numerical complexity, where $m_{local}$ is the number of obstacles within the local space, and $m_{ref}$ is the reference number (i.e., the total number of obstacles); $C_{h}$ represents the historical sampling complexity, where $N_{fail,recent}$ is the number of failures (collisions or invalid expansions) in the recent L sampling attempts, and $N_{total,recent}$ is the total number of attempts in the recent L sampling iterations (in this paper, L=20, meaning the history of the most recent 20 sampling attempts is considered); and $\lambda_{1}$, $\lambda_{2}$, and $\lambda_{3}$ are the weighting coefficients, set as $\lambda_{1}=2$, $\lambda_{2}=1.5$, and $\lambda_{3}=1$.

The dynamic initialization formula is

(10) $$\left\{\begin{array}{l} b_{0}=b_{\mathrm{init}}=\frac{a^{\prime }}{2}\cdot \exp\left[-\left(2\cdot \mathrm{vol}\_\mathrm{ratio}+\frac{\mathrm{obstacle}\_\mathrm{count}}{\mathrm{total}\_\mathrm{obstacles}}\right)\right]\\ a_{0}^{\prime }=a^{\prime } \end{array}\right.$$

In the equation, vol_ratio is the ratio of the total volume of obstacles contained within the local space to the total volume of the entire space, obstacle_count is the number of obstacles contained within the local space, total_obstacles is the total number of obstacles in the environment, and $b_{\mathrm{init}}$ is the dynamically initialized initial value of the ellipsoid’s semi-minor axis.

The local space is defined as a spherical region centered at $q_{\mathrm{new}}$ with radius $r^{\prime }$. The specific formula is as follows:

(11) $$r_{k}^{\prime }=k_{1}\cdot a_{k}^{\prime }$$

Here, $k_{1}$ is a proportional coefficient; it is recommended to set $0.5\leq k_{1}\leq 1.0$ (with $k_{1}$ being approximately 0.7).

When the semi-major axis $a_{k}^{\prime }$ of the ellipsoid grows exponentially, $r_{k}^{\prime }$ expands synchronously, ensuring that the local region around $q_{\mathrm{new}}$ always remains appropriately matched to the scale of the ellipsoid. The dynamic $r^{\prime }$ can more accurately match the local environmental requirements around $q_{\mathrm{new}}$.

#### 4.1.3. Mathematical Principle of Ellipsoidal Sampling

The fundamental principle of ellipsoidal sampling is to sequentially apply scaling, rotation, and translation transformations to points within a unit sphere; these points are mapped into the interior space of the ellipsoid.

To obtain uniformly distributed random points within the unit sphere, uniform sampling in spherical coordinates can be employed: first generate a random azimuthal angle θ, polar angle φ, and radius r; then, convert the spherical coordinates to Cartesian coordinates (x,y,z).

Generating random point coordinates in the spherical coordinate system, i.e.,

(12) $$\left\{\begin{array}{c} \theta ={\mathrm{rand}}^{\prime }(1)\times 2\pi \\ \varphi ={\mathrm{rand}}^{\prime }(1)\times \pi \\ r=\sqrt[{3}]{{\mathrm{rand}}^{\prime }(1)} \end{array}\right.$$

In the equation, ${\mathrm{rand}}^{\prime }(1)$ is a function that generates a uniformly distributed random number within the interval [0, 1].

The transformation from spherical coordinates to Cartesian coordinates is given by

(13) $$\left\{\begin{array}{c} x=r\times \sin(\varphi )\cos(\theta )\\ y=r\times \sin(\varphi )\sin(\theta )\\ z=r\times \cos(\varphi ) \end{array}\right.$$

The coordinate transformation process is as follows:

(1) Generate a local coordinate system, i.e.,

(14) $$\left\{\begin{array}{cc} \;\;u=\frac{q_{\mathrm{goal}}-q_{\mathrm{init}}}{\left\|q_{\mathrm{goal}}-q_{\mathrm{init}}\right\|}\\ v=\left\{\begin{array}{c} \begin{array}{cc} \;\;{\left[1,0,0\right]}^{T} & \;\;\;\;\left\|u_{xy}\right\|<\varepsilon \end{array}\\ \begin{array}{cc} \frac{{\left[-u_{y},u_{x},0\right]}^{T}}{\sqrt{{u_{x}}^{2}+{u_{y}}^{2}}} & \;\;\mathrm{otherwise} \end{array} \end{array}\right.\\ w=u\times v & \end{array}\right.$$

In the equation, u, v, and w are basis vectors; u is a unit vector, obtained by normalizing the vector from the start point $q_{\mathrm{init}}$ to the goal point $q_{\mathrm{goal}}$, i.e., $q_{\mathrm{goal}}-q_{\mathrm{init}}$; $u_{y}$ is the component of u in the y-direction; $u_{x}$ is the component of u in the x-direction; $u_{xy}$ is the projection of vector u onto the xy-plane; ε is a predefined small positive number, representing a threshold value; and vector w is obtained by the cross product of vector u and vector v.

(2) Construct the rotation matrix R, i.e.,

(15) $$R=\left[u|v|w\right]\in SO(3)$$

In the equation, SO(3) denotes the three-dimensional special orthogonal group.

(3) Generate the sample point, i.e.,

(16) $$P_{\mathrm{global}}=R\cdot \left[\begin{array}{c} a^{\prime }\cdot x\\ b\cdot y\\ c\cdot z \end{array}\right]+\mathrm{center}$$

In the equation, $x^{2}+y^{2}+z^{2}\leq 1$, c is the semi-axis length of the line segment passing through point M and perpendicular to both the semi-major axis $a^{\prime }$ and semi-minor axis b of the ellipsoid, and center is the translation vector representing the center of the ellipsoid.

#### 4.1.4. Analysis of the Dynamic Adjustment Mechanism

For the semi-major axis $a^{\prime }$, the adjustment strategy is to grow exponentially with the number of iterations, thereby accelerating the search. The formula is $a_{k}^{\prime }=a_{0}^{\prime }{\left(1+1/n\right)}^{k/m}$, where m is the threshold for adjusting the ellipsoid every m iterations.

For the semi-minor axis b, the adjustment strategy is that its initial value is determined by environmental complexity, and thereafter, it grows synchronously with the growth rate of $a^{\prime }$. The formula is $b_{k}=b_{\mathrm{init}}{\left(1+1/n\right)}^{k/m}$, where m is the threshold for adjusting the ellipsoid every m iterations.

For the aspect ratio (shape ratio), the adjustment strategy is that the initial ellipsoid eccentricity is positively correlated with obstacle distribution. The formula is $\mathrm{flatness}=((a^{\prime }-b)/a^{\prime })\propto \exp(\mathrm{obstacle}\_\mathrm{density})$, where flatness is the initial ellipsoid flatness (eccentricity), and obstacle_density is the obstacle density.

#### 4.1.5. Analysis of the Environmental Perception Mechanism

(1) The obstacle influence factor complexity_factor is
  (17) $$\mathrm{complexity}\_\mathrm{factor}=-\left(2\cdot \mathrm{vol}\_\mathrm{ratio}+2\cdot \frac{\mathrm{obstacle}\_\mathrm{count}}{\mathrm{total}\_\mathrm{obstacles}}\right)$$
(2) The mechanism of action is as follows:

For the vol_ratio term, the greater the proportion of total obstacle volume within the local space, the flatter the ellipsoid (i.e., the smaller b), which enhances axial search along the primary direction. For the obstacle_count term, the more obstacles intersected by the path, the flatter the ellipsoid, which avoids densely clustered obstacle regions.

#### 4.1.6. Algorithm Characteristics Analysis

(1) Directed search: The major axis always points toward the target direction, accelerating the process of approaching the goal;
(2) Obstacle avoidance: Employs a dynamic contraction mechanism for the semi-minor axis b, automatically narrowing the search width in regions with dense obstacles;
(3) Progressive optimality: The ellipsoid volume grows exponentially to balance explora-tion and exploitation, gradually covering the entire configuration space;
(4) Environmental adaptation: Based on real-time collision detection feedback, dynami-cally adjusts the sampling strategy according to the actual distribution of obstacles.

The dynamic ellipsoidal sampling strategy focuses the search initially on the region between the start and goal points and then gradually expands the search scope as the number of iterations increases and the proportion of obstacle volume decreases. If a feasible path remains difficult to find over a long period, the search will eventually cover the entire space, thereby significantly improving the likelihood of discovering challenging paths.

### 4.2. Dynamic Goal-Biased Strategy

The dynamic goal-biased strategy guides the random tree toward the goal in a greedy manner, thereby reducing redundant exploration caused by random sampling and improving path planning efficiency.

The dynamic goal-biased strategy consists of the following steps:

(1) Calculate the direction vector. Given the current nearest node $q_{\mathrm{near}}$ and the goal point $q_{\mathrm{goal}}$, the direction vector d is defined as their difference, i.e.,
  (18) $$d=q_{\mathrm{goal}}-q_{\mathrm{near}}$$
(2) Adjust the step size $d_{\mathrm{unit}}$. If the magnitude (norm) of the direction vector exceeds the step size η, normalize it and then multiply by η; otherwise, use the direction vector directly, i.e.,
  (19) $$d_{\mathrm{unit}}=\left\{\begin{array}{cc} \frac{d}{\left\|d\right\|}\cdot \eta & \;\left\|d\right\|>\eta \\ d & \mathrm{otherwise} \end{array}\right.$$
(3) Generate the new node, i.e.,
  (20) $$q_{\mathrm{new}}=q_{\mathrm{near}}+d_{\mathrm{unit}}$$

The path must satisfy collision-free and safety distance constraints: The path does not intersect any obstacles, $l(q_{\mathrm{new}},obstacles)>l_{\min}$.

During the algorithm expansion process, the dynamic goal-biased strategy prioritizes extending the path toward the target point $q_{\mathrm{goal}}$, i.e., based on the current node $q_{\mathrm{near}}$, it attempts to generate a new node $q_{\mathrm{new}}$ in the direction of the goal. If the target point is relatively far away, the node is extended by the predefined step size η; if the target point lies within the neighborhood, an attempt is made to establish a direct connection. All candidate path segments must pass collision detection and safety distance validation to ensure the generated path is safe and feasible. In obstacle-free environments, this strategy helps rapidly converge the path toward the goal, significantly improving search efficiency. In complex obstacle environments, however, the strategy must be combined with artificial potential field repulsive forces to assist in obstacle avoidance or leverage a reconnection mechanism to adjust the path direction and avoid local deadlock. The advantage of this strategy lies in its ability to effectively compress ineffective search space and accelerate the generation of feasible paths. Its drawback is that, when facing narrow passages or densely clustered obstacle regions, it may become trapped in local optima; therefore, it typically needs to be supplemented with random sampling or potential field guidance for additional exploration.

To address the issue that the dynamic target bias strategy is prone to falling into local optima in narrow passages or dense obstacle environments, this paper integrates it with the improved artificial potential field strategy described in Section 4.5 for cooperative guidance. The specific collaborative mechanism is as follows:

(1) Direction vector synthesis:

When generating a new node, not only is the attraction in the direction of the target point considered, but the resultant force direction calculated by the artificial potential field is also simultaneously introduced. Let the target bias direction vector be $d_{\mathrm{bias}}$ (Equation (20)) and the resultant force direction of the artificial potential field be $d_{\mathrm{apf}}$ (Equation (36)). Then, the final extension direction $d_{\mathrm{final}}$ is obtained through weighted fusion as follows:

(21) $$d_{\mathrm{final}}=\alpha_{2}\cdot d_{\mathrm{bias}}+\beta_{2}\cdot d_{\mathrm{apf}}$$

where $\alpha_{2}$ and $\beta_{2}$ are the weighting coefficients for the target bias and potential field guidance, respectively, satisfying $\alpha_{2}+\beta_{2}=1$. Initially, they are set as $\alpha_{2}=0.7$ and $\beta_{2}=0.3$ to maintain strong target orientation. When it is detected that the path falls into local oscillation or repeated collisions, the coefficients are dynamically adjusted to $\alpha_{2}=0.4$ and $\beta_{2}=0.6$ to enhance the obstacle avoidance effect of the potential field.

(2) Local minimum escape strategy:

If consecutive $N_{stuck}$ attempts (in this paper, $N_{stuck}=5$) at expansion fail due to collisions, the algorithm is judged to have fallen into a local minimum. At this point, the target bias is temporarily abandoned, and a pure artificial potential field-guided “escape exploration” is performed for several steps (e.g., 10 steps) until the local region is exited, after which the fusion strategy is restored.

Through the aforementioned collaborative mechanism, the dynamic target bias strategy effectively utilizes potential field information to avoid dense obstacles and narrow passages while maintaining efficient convergence toward the target, thereby significantly reducing the occurrence of local optima.

### 4.3. Heuristic Nearest Neighbor Selection Strategy

The heuristic nearest neighbor selection strategy accelerates path planning convergence by dynamically balancing heuristic guidance and random exploration.

This heuristic nearest neighbor selection strategy consists of two parts:

(1) Optimal node selection based on a heuristic function.

When selecting a heuristic node with probability $p_{heuristic}$, the node with the smallest F value is chosen as the nearest neighbor.

(22) $$q_{nearest}=\arg\;\underset{q\in Nodes}{\min}(F(q))$$

where

(23) $$F(q)=\underset{\mathrm{path}\;\mathrm{cumulative}\;\mathrm{cost}}{\underset{︸}{G(q)}}+\underset{\mathrm{heuristic}\;\mathrm{estimate}}{\underset{︸}{H(q,q_{\mathrm{goal}})}}$$

G(q) represents the actual path cost from the start point to node q (e.g., path length), and $H(q,q_{\mathrm{goal}})$ represents the heuristic function, typically the Euclidean distance $\left\|q-q_{\mathrm{goal}}\right\|$.

(2) Nearest neighbor selection for randomly sampled points.

When selected with probability $1-p_{heuristic}$, directly find the node that is geometrically closest to the random sample point $q_{\mathrm{rand}}$:

(24) $$q_{nearest}=\arg\;\underset{q\in Nodes}{\min}(\left\|q-q_{\mathrm{rand}}\right\|)$$

The heuristic nearest neighbor selection strategy must satisfy the following constraints:

(1) Probability weight adjustment.

The heuristic selection probability $p_{heuristic}$ is dynamically adjusted according to the number of iterations:

(25) $$p_{heuristic}=0.1+\frac{iter}{\max\_iter}$$

In the equation, iter represents the current iteration count, and max_iter represents the maximum number of iterations. In the early stages, the strategy favors random exploration (smaller $p_{heuristic}$); in later stages, it shifts toward heuristic optimization.

(2) Path feasibility verification.

Regardless of which nearest neighbor is selected, the new node $q_{\mathrm{new}}$ must satisfy: The path from $q_{\mathrm{nearest}}\to q_{\mathrm{new}}$ to must not intersect any obstacles, $l(q_{\mathrm{new}},obstacles)>l_{\min}$.

The heuristic nearest neighbor selection strategy consists of two components: heuristic guidance and random exploration. When the heuristic selection probability $p_{heuristic}$ is large, the strategy favors heuristic selection (choosing the node with the smallest F value), guiding the tree to grow toward the goal direction, reducing redundant branches, and emphasizing local optimization (shortening path length). When $p_{heuristic}$ is small (i.e., $1-p_{heuristic}$ is large), the strategy favors random exploration (selecting the geometrically nearest node), maintaining exploration capability, avoiding getting trapped in local optima, and emphasizing global exploration (preventing feasible paths from being missed).

### 4.4. Random Sampling Expansion Strategy

By using random sampling to guide the tree to expand into unexplored regions, this approach avoids getting trapped in local minima while ensuring global search capability.

The expansion of random sample points consists of the following steps:

(1) Random sample point generation.

Uniformly randomly sample a point $q_{\mathrm{rand}}$ in the configuration space C:

(26) $$q_{\mathrm{rand}}=\left[x_{\mathrm{rand}},y_{\mathrm{rand}},z_{\mathrm{rand}}\right],where\;x_{\mathrm{rand}}∼U(0,x_{\max}),y_{\mathrm{rand}}∼U(0,y_{\max}),z_{\mathrm{rand}}∼U(0,z_{\max})$$

Here, U(a,b) represents a uniformly distributed random variable within the interval $\left[a,b\right]$, and $x_{\max},y_{\max},z_{\max}$ represent the spatial dimensions of the environment.

(2) Nearest neighbor node selection.

From all nodes in the tree, select the node $q_{\mathrm{nearest}}$ that is geometrically closest to $q_{\mathrm{rand}}$:

(27) $$q_{\mathrm{nearest}}=\arg\;\underset{q\in Nodes}{\min}(\left\|q-q_{\mathrm{rand}}\right\|)$$

$\left\|\cdot \right\|$ represents the Euclidean distance.

(3) Direction vector calculation.

Generate a unit direction vector from $q_{\mathrm{nearest}}$ to $q_{\mathrm{rand}}$ and extend it by the step size η:

(28) $$d^{\prime }=q_{\mathrm{rand}}-q_{\mathrm{nearest}},d_{\mathrm{unit}}^{\prime }=\frac{d^{\prime }}{\left\|d^{\prime }\right\|},q_{\mathrm{new}}=q_{\mathrm{nearest}}+\eta \cdot d_{\mathrm{unit}}^{\prime }$$

If $\left\|d^{\prime }\right\|<\eta$, then directly take $q_{\mathrm{rand}}$ as the new node $q_{\mathrm{new}}$.

(4) Path feasibility verification.

The new node $q_{\mathrm{new}}$ must satisfy the following conditions: The path from $q_{\mathrm{nearest}}\to q_{\mathrm{new}}$ to must not intersect any obstacles, $l(q_{\mathrm{new}},obstacles)>l_{\min}$.

The random sampling expansion strategy is probabilistically complete: if a feasible path exists, it will be found with probability 1 within the maximum number of iterations. It balances exploration and exploitation—the randomness enables exploration of new regions, while the nearest neighbor strategy promotes local path optimization. Random sample points are generated using uniform sampling, which ensures candidate points are uniformly distributed over the entire configuration space, guaranteeing coverage of the entire free space. In complex environments (e.g., narrow passages), improved sampling strategies such as ellipsoidal sampling or Gaussian sampling can be adopted to enhance performance. By selecting the node in the tree nearest to the random sample point as the expansion base, the strategy ensures locally shortest path extensions and reduces time complexity. The step size η controls the expansion speed: a smaller step size improves path accuracy but increases the number of iterations, whereas a larger step size may skip over narrow obstacles.

### 4.5. Improved Artificial Potential Field Strategy

By integrating the local obstacle avoidance capability of artificial potential fields with the global optimality of the RRT* algorithm, this approach addresses the problem of low planning efficiency exhibited by traditional RRT in environments with dense obstacles.

The core of the fusion strategy between artificial potential fields and the RRT* algorithm lies in computing the node expansion direction through a combination of the attractive force (guiding toward the goal) and repulsive force (avoiding obstacles) from the artificial potential field. The steps are as follows:

Compute the total direction vector $d_{\mathrm{total}}$, i.e.,

(29) $$d_{\mathrm{total}}=F_{\mathrm{att}}+F_{\mathrm{rep}}+F_{\mathrm{near}}$$

In the equation, $F_{\mathrm{att}}$ is the combined attractive force between the goal point and the random point, $F_{\mathrm{rep}}$ is the repulsive force generated by obstacles, and $F_{\mathrm{near}}$ is the repulsive force generated by neighboring nodes (to prevent node clustering).

The formula for the attractive force is as follows.

The attractive force consists of a weighted direction combining the goal point and the random sample point, i.e.,

(30) $$F_{\mathrm{att}}=\alpha_{1}^{\prime }\cdot \frac{q_{\mathrm{goal}}-q_{\mathrm{current}}}{\left\|q_{\mathrm{goal}}-q_{\mathrm{current}}\right\|}+\beta_{1}\cdot \frac{q_{\mathrm{rand}}-q_{\mathrm{current}}}{\left\|q_{\mathrm{rand}}-q_{\mathrm{current}}\right\|}$$

In the equation, $\alpha_{1}^{\prime }$ and $\beta_{1}$ are the attractive force weights for the goal point and the random point, respectively; $q_{\mathrm{current}}$ is the coordinate of the current node; $q_{\mathrm{goal}}$ is the goal point; and $q_{\mathrm{rand}}$ is the random point.

The repulsive force from obstacles is divided into a basic repulsive force $F_{\mathrm{rep}1}$ and an additional repulsive force $F_{\mathrm{rep}2}$ (to avoid local minima), i.e.,

Repulsive force formula (for obstacles) is as follows:

(31) $$F_{\mathrm{rep}}=\sum_{obs}\left(F_{\mathrm{rep}1}+F_{\mathrm{rep}2}\right)$$

The basic repulsive force (repulsion effect) is

(32) $$F_{\mathrm{rep}1}=k_{\mathrm{rep}}\left(\frac{1}{d_{\mathrm{obs}}}-\frac{1}{d_{\mathrm{rep}\_\mathrm{range}}}\right)\cdot \frac{\rho_{g}^{2}}{d_{\mathrm{obs}}^{2}}\cdot d_{\mathrm{obs}}$$

The additional repulsive force (goal-oriented correction) is

(33) $$F_{\mathrm{rep}2}=\frac{n_{1}}{2}k_{\mathrm{rep}}{\left(\frac{1}{d_{\mathrm{obs}}}-\frac{1}{d_{\mathrm{rep}\_\mathrm{range}}}\right)}^{2}\cdot \rho_{g}^{n-1}\cdot \frac{q_{\mathrm{current}}-q_{\mathrm{goal}}}{\rho_{g}}$$

In the equation, $k_{\mathrm{rep}}$ is the obstacle repulsion constant, $n_{1}$ is the control exponent for repulsive force, governing its attenuation, $d_{\mathrm{obs}}$ is the distance from the current node to the obstacle surface, $d_{\mathrm{rep}\_\mathrm{range}}$ is the effective range of the repulsive force, $\rho_{g}=\left\|q_{\mathrm{current}}-q_{\mathrm{goal}}\right\|$ is the distance from the current node to the goal, and $d_{\mathrm{obs}}$ is the unit direction vector pointing from the obstacle surface toward the current node.

The node repulsion formula (to prevent clustering) is as follows:

(34) $$F_{\mathrm{near}}=\eta_{\mathrm{repel}}\cdot \sum_{d_{\mathrm{node}}<r_{4}}\frac{d_{\mathrm{node}}}{d_{\mathrm{node}}^{2}}$$

In the equation, $\eta_{\mathrm{repel}}$ is the node repulsion constant, $d_{\mathrm{node}}=\left\|q_{\mathrm{current}}-q_{\mathrm{node}}\right\|$ is the distance between the current node and other nodes, $r_{4}$ is the radius of influence for the repulsive force, and $d_{\mathrm{node}}$ is the unit direction vector pointing from other nodes toward the current node.

After combining all directional components, normalize the resulting vector and generate the candidate node, i.e.,

(35) $$d_{\mathrm{total}}^{\prime }=\frac{d_{\mathrm{total}}}{\left\|d_{\mathrm{total}}\right\|}$$

(36) $$q_{\mathrm{new}}=q_{\mathrm{current}}+\eta \cdot d_{\mathrm{total}}^{\prime }$$

In the equation, η is the expansion step size, $d_{\mathrm{total}}^{\prime }$ is the coordinate of the candidate node, $d_{\mathrm{total}}$ is the total direction vector, and $\left\|d_{\mathrm{total}}\right\|$ is the magnitude (norm) of the total direction vector.

### 4.6. Reconnection Optimization Strategy

Through local reconnection optimization, the cumulative path cost of new nodes is reduced while balancing path length, smoothness, and safety. The reconnection strategy selects a better parent node for the new node by searching neighboring nodes and optimizing based on multi-objective cost.

The reconnection optimization strategy includes the following steps:

(1) Cumulative path cost.

The total path length from the root node (start point) to node $q_{i}$ is

(37) $$C_{path}(q_{i})=\sum_{k=1}^{m}\left\|q_{k}-q_{k-1}\right\|$$

In the equation, $C_{path}(q_{i})$ represents the total path length from the root node (start point) to node $q_{i}$, $q_{0}$ is the root node, and $q_{m}=q_{i}$.

It can also be computed recursively:

(38) $$C_{path}(q_{i})=C_{path}(q_{parent(i)})+\left\|q_{i}-q_{parent(i)}\right\|$$

(2) Comprehensive cost function.

When the new node $q_{\mathrm{new}}$ selects a parent node $q_{j}$, the comprehensive cost is

(39) $$C_{total}(q_{j})=\underset{\mathrm{base}\;\mathrm{path}\;\mathrm{cost}}{\underset{︸}{C_{path}(q_{j})+\left\|q_{j}-q_{\mathrm{new}}\right\|}}+\underset{\mathrm{turning}\;\mathrm{angle}\;\mathrm{penalty}}{\underset{︸}{\beta_{2}\cdot \theta^{\prime }(q_{\mathrm{grand}},q_{j},q_{\mathrm{new}})}}+\underset{\mathrm{obstacle}\;\mathrm{distance}\;\mathrm{penalty}}{\underset{︸}{\beta_{3}\cdot e^{-l_{\mathrm{obs}}}}}$$

In the equation, $C_{total}(q_{j})$ represents the total path length from the root node (start point) to node $q_{j}$; $\beta_{2}$ represents the weight controlling the turning angle penalty term; $\beta_{3}$ represents the weight controlling the obstacle distance penalty term; $\theta^{\prime }$ represents the path turning angle (in radians), formed by the grandparent node $q_{\mathrm{grand}}$, the candidate parent node $q_{j}$, and the new node $q_{\mathrm{new}}$; and $l_{\mathrm{obs}}$ represents the minimum distance from the path segment $q_{j}\to q_{\mathrm{new}}$ to obstacles.

(3) Turning angle penalty calculation.

The path turning angle is defined by the angle between vectors:

(40) $$\theta^{\prime }=\arccos(\frac{v_{1}\cdot v_{2}}{\left\|v_{1}\right\|\cdot \left\|v_{2}\right\|})$$

In the equation, $v_{1}=q_{j}-q_{\mathrm{grand}}$, $v_{2}=q_{\mathrm{new}}-q_{j}$, and the penalty term $\beta_{2}\cdot \theta^{\prime }$ encourages smoother paths and helps avoid sharp turns. Since $\left\|v_{1}\right\|\cdot \left\|v_{2}\right\|$ may become zero, to ensure the numerical stability of the angle calculation, the algorithm constrains the minimum step length during node expansion to avoid node overlap. Additionally, when computing $\theta^{\prime }$, a denominator protection mechanism is adopted: if $\left\|v_{1}\right\|\cdot \left\|v_{2}\right\|<\varepsilon^{\prime }$ (where $\varepsilon^{\prime }$ is an extremely small positive number, such as $10^{-10}$), $\theta^{\prime }$ is directly set to 0. In practical calculations, the denominator term is replaced by $\max(\left\|v_{1}\right\|\cdot \left\|v_{2}\right\|,\varepsilon^{\prime })$ to avoid division-by-zero errors.

(4) Obstacle distance penalty.

The minimum distance $l_{\mathrm{obs}}$ between the path segment and obstacles is mapped to a penalty term via an exponential function:

(41) $$Penalty_{\mathrm{obs}}=\beta_{2}\cdot e^{-l_{\mathrm{obs}}}$$

In the equation, $Penalty_{\mathrm{obs}}$ represents the obstacle distance penalty. When $l_{\mathrm{obs}}\to 0$ (approaching an obstacle), the penalty approaches $\beta_{2}$. When $l_{\mathrm{obs}}>l_{\min}$, the penalty can be neglected (safety distance is satisfied).

(5) Parent node selection condition.

Select the node with the minimum comprehensive cost within the neighborhood as the parent node:

(42) $$q_{parent}=\arg\;\underset{q_{j}\in N_{\mathrm{radius}}}{\min}(C_{total}(q_{j}))$$

In the equation, $N_{\mathrm{radius}}=\left\{q_{j}|\left\|q_{j}-q_{\mathrm{new}}\right\|<r_{3}\right\}$, where $r_{3}$ is the neighborhood radius.

The path must satisfy collision-free and safety distance constraints: The path from $q_{\mathrm{nearest}}\to q_{\mathrm{new}}$ to must not intersect any obstacles, $l(q_{\mathrm{new}},obstacles)>l_{\min}$.

The reconnection optimization strategy restricts the search scope via the neighborhood radius, reducing computational load. The base path cost directly optimizes path length; the turning angle penalty reduces unnecessary turns in the path, enhancing motion feasibility (e.g., respecting UAV turning radius limits); and the obstacle distance penalty encourages paths to stay farther from obstacles, improving the safety margin while enforcing a safety distance constraint $l_{\mathrm{obs}}>l_{\min}$ to prevent paths from closely hugging obstacles.

### 4.7. Path Pruning Strategy

**Figure 8:**
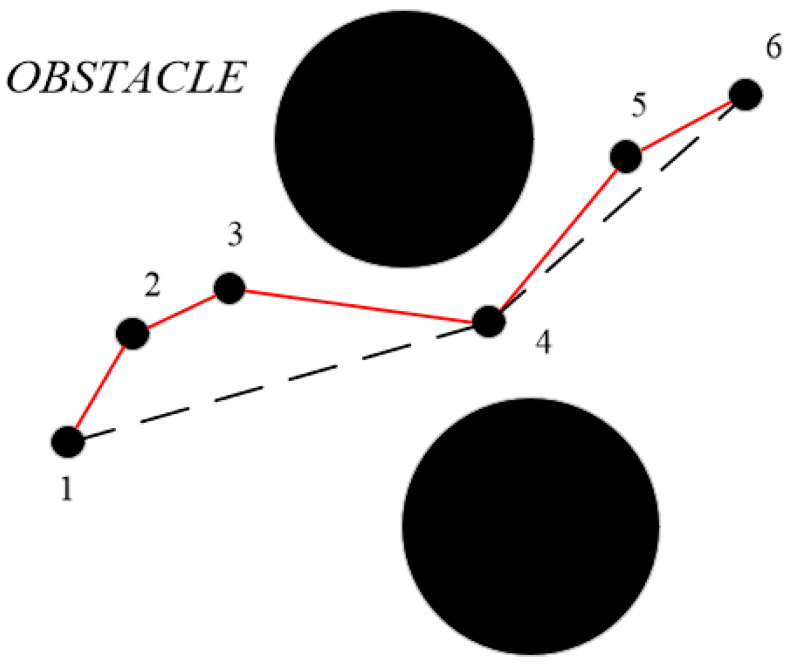
Path pruning principle diagram. The small black dots represent path nodes, the red solid line represents the path before pruning, and the black dashed line represents the path after pruning.

After optimization using the aforementioned strategies, redundant nodes in the path are significantly reduced. Based on this, further path pruning is applied for final optimization. As shown in Figure 8, the pruning principle is as follows: traverse the random tree path from either the start or end point and remove redundant nodes to obtain a shorter feasible path. In Figure 8, the original path is represented by the red solid line segment. From point 1 to points 2, 3, and 4, no collisions occur; however, a collision occurs when reaching point 5. Therefore, point 1 is directly connected to the node preceding point 5 (i.e., point 4). Taking point 4 as the base, since no collisions occur between point 4 and points 5 or 6, point 4 is connected directly to point 6. The optimized path segments are shown as black dashed lines in the figure. Points 2, 3, and 5 are redundant nodes.

### 4.8. Path Smoothing

Although the pruned path is shortened, it introduces numerous sharp bends, resulting in a zigzag trajectory with frequent undulations. Such a path not only makes it difficult to ensure smooth operation of the robotic arm but also accelerates mechanical wear and shortens its service life. Therefore, path smoothing is crucial. In this paper, a smoothing method based on cubic B-spline basis functions is adopted to optimize the pruned path.

The formula for the cubic B-spline curve B(t) is as follows:

(43) $$B(t)=\sum_{i=0}^{3}B_{i,k}(t)P_{i}$$

(44) $$\begin{array}{l} B_{0,3}(t)=\frac{1}{6}{\left(1-t\right)}^{3},\;B_{1,3}(t)=\frac{1}{6}(3t^{3}-6t^{2}+4)\\ B_{2,3}(t)=\frac{1}{6}(-3t^{3}+3t^{2}+3t+1),\;B_{3,3}(t)=\frac{1}{6}t^{3},\;t\in \left[0,1\right] \end{array}$$

**Figure 9:**
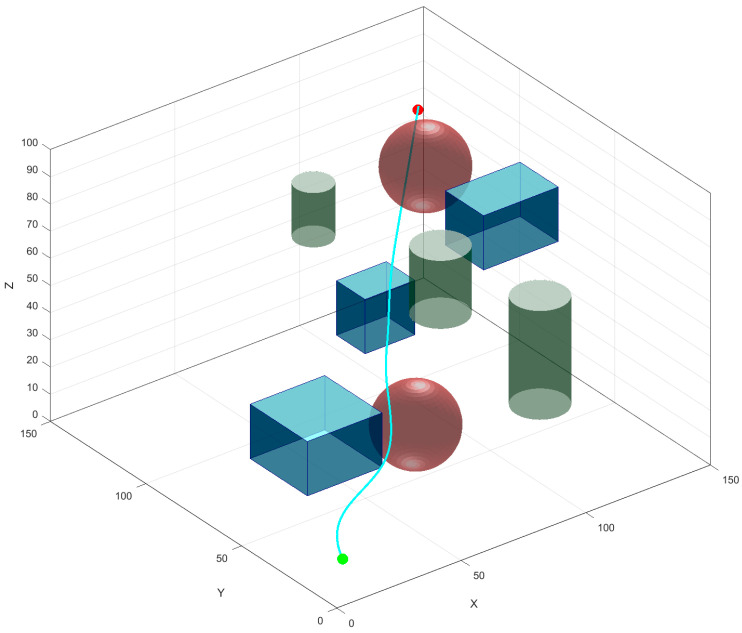
Path smoothing effect. Cubes, cuboids, spheres, and cylinders represent obstacles, and the blue solid line represents the optimized path.

In the equation, $P_{i}$ represents the control points of the curve, and $B_{i,k}(t)$ represents the cubic B-spline basis functions. During the smoothing process, the specific coordinates of the control points $P_{i}$ are first determined. Then, by substituting these control points and basis functions into Equation (43) according to Equation (44), the coordinates of all points conforming to the definition of the cubic B-spline curve can be computed. The effect of path smoothing is shown in Figure 9.

### 4.9. Algorithm Flow

**Figure 10:**
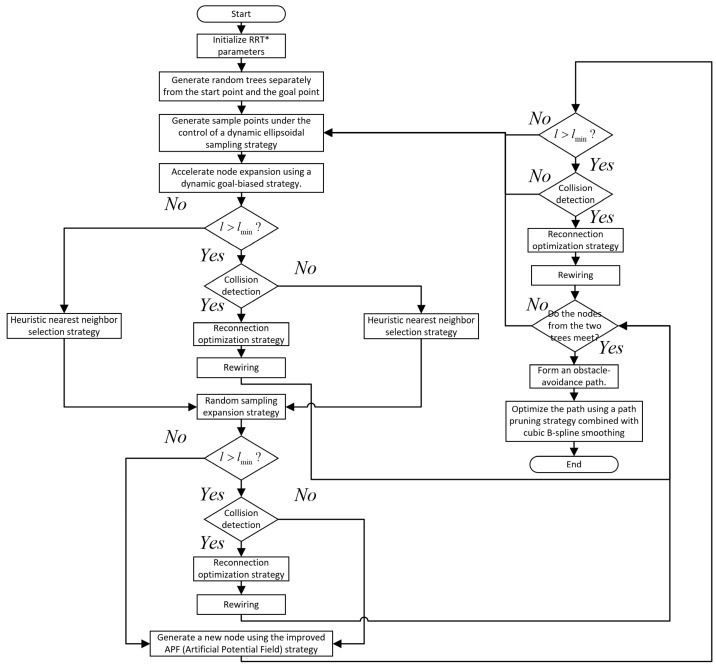
Algorithm flow.

The algorithm flow of this paper is shown in Figure 10.

Step 1: Initialize the environment and algorithm parameters and construct two separate random expansion trees rooted at the start point and the goal point, respectively.

Step 2: Employ a dynamic elliptical sampling strategy to generate sample points. Node expansion utilizes a dynamic goal-biasing strategy to enhance search efficiency and speed. Evaluate the relationship between l (distance from the new node to obstacles) and $l_{\min}$ (a predefined threshold). If $l>l_{\min}$, proceed to collision detection. Otherwise, direct the process to heuristic nearest neighbor selection to generate the nearest neighbor for both random sampling expansion and improved APF, then proceed with random sampling expansion. If a collision is detected, redirect to heuristic nearest neighbor selection for both strategies and resume random sampling expansion. If no collision occurs, perform reconnection optimization and rewiring, then determine whether the two random search trees have met. If they meet, a collision-free path is formed. Otherwise, resample and continue.

Step 3: Introduce the random sampling expansion strategy. Again, evaluate the relationship between l and $l_{\min}$. If $l>l_{\min}$, proceed to collision detection. Otherwise, direct the process to the improved APF strategy. If a collision is detected, redirect to the improved APF strategy. If no collision occurs, perform reconnection optimization and rewiring, then check whether the two random search trees have met. If they meet, a collision-free path is formed. Otherwise, resample and continue.

Step 4: Introduce the improved artificial potential field (APF) strategy. Compute the total direction vector to determine the position of the new node. Evaluate the relationship between l and $l_{\min}$. If $l>l_{\min}$, proceed to collision detection. Otherwise, resample. If a collision is detected, resample. If no collision occurs, perform reconnection optimization and rewiring.

Step 5: Determine whether the two newly generated nodes (one from each tree) have met. If not, continue iterating. If they meet, proceed to the next step.

Step 6: Merge the path points, apply the path pruning strategy combined with cubic B-spline smoothing for final path optimization, and conclude the robotic arm obstacle-avoidance path planning algorithm.

## 5. Obstacle-Avoidance Path Planning Simulation

To validate the proposed improved RRT* algorithm, simulation experiments were conducted for the following algorithms: the standard RRT*, GB-RRT* (goal-biased RRT*), BI-RRT* (bidirectional RRT*), APF-RRT* (artificial potential field–RRT*), BI-APF-RRT*, and the improved RRT* algorithm. The simulations were performed on a system equipped with a 13th Gen Intel^®^ Core™ i7-13620H processor running at 2.40 GHz and 16.0 GB of RAM, Lenovo computer manufacturer, Tianjin, China. The 3D simulation environment was configured with dimensions of 150 cm × 150 cm × 100 cm. The start and goal points were set at coordinates (10 cm, 10 cm, 20 cm) and (140 cm, 140 cm, 80 cm), respectively. The environment contained various irregularly shaped obstacles of different sizes, primarily including cubes, cuboids, spheres, and cylinders. Each algorithm underwent 30 independent path planning simulation trials. In the resulting figures, the start and goal points are marked by light-colored and dark-colored spheres, respectively, for clear distinction, and the final planned paths from each algorithm are shown as solid lines.

### 5.1. Simulation and Analysis in a Simple Environment

**Figure 11:**
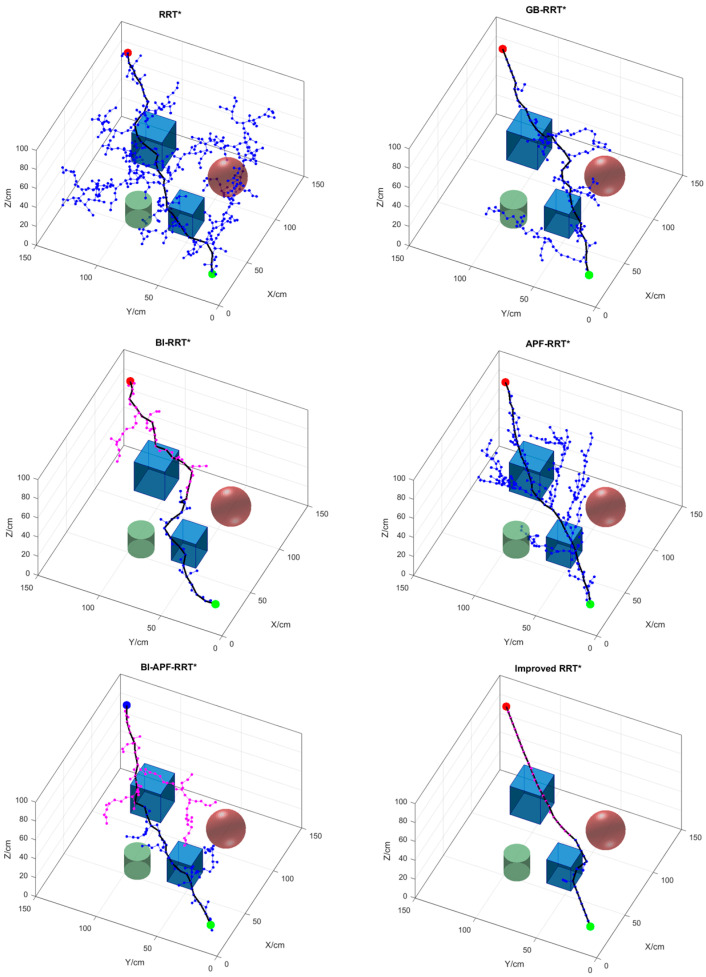
Obstacle-avoidance path planning results of the six algorithms in a simple environment. The green sphere represents the starting point (10 cm, 10 cm, 20 cm), the red sphere represents the goal point (140 cm, 140 cm, 80 cm), the light red and purple points represent the sampling points, the cube, cuboid, sphere, and cylinder represent obstacles, and the solid lines represent the final paths planned by each algorithm.

Figure 11 shows the obstacle-avoidance path planning results of six different algorithms in a simple environment, and their comparisons are summarized in Table 2. As can be seen from Figure 11, due to the large sampling space of RRT*, its resulting trajectory contains more turning points and a longer path. Compared with the original RRT* algorithm, GB-RRT* exhibits faster growth of the random tree, requires less planning time, and produces a shorter path. Although GB-RRT* improves search efficiency, it lacks obstacle repulsion, leading to redundant nodes around obstacles. In contrast to GB-RRT*, BI-RRT* switches from unidirectional to bidirectional search, significantly reducing the number of path nodes. Compared with BI-RRT*, APF-RRT* introduces obstacle repulsion, further enhancing search efficiency. Moreover, BI-APF-RRT* improves upon APF-RRT* by adopting bidirectional search, substantially decreasing both the average planning time and the average number of path nodes. The improved RRT* algorithm proposed in this paper demonstrates superior performance in terms of average planning time, average number of path nodes, and average path length.

By analyzing the data in Table 2, in the simple map environment, the improved RRT* algorithm reduces the planning time by 90.57%, 58.33%, 65.52%, 84.85%, and 78.26% compared to RRT*, GB-RRT*, BI-RRT*, APF-RRT*, and BI-APF-RRT*, respectively. The number of path nodes is reduced by 91.96%, 67.84%, 40.22%, 87.18%, and 53.39%, respectively. The path length is shortened by 17.36%, 11.94%, 8.74%, 8.09%, and 9.11%, respectively. The improved RRT* algorithm proposed in this paper significantly enhances performance in 3D environments: both planning time and the number of redundant nodes are substantially reduced, and the resulting obstacle-avoidance paths are smoother.

To better analyze the stability of the six algorithms, 30 experiments were conducted. Based on data analysis, the variance and standard deviation of the planning time, number of path nodes, and path length for the six algorithms across the 30 experiments are shown in Table 3, Table 4 and Table 5, respectively.

Based on the data analysis from Table 3, Table 4 and Table 5, the six algorithms demonstrate varying performance in planning time, number of path nodes, and path length within a simple map environment. Specifically, the variance and standard deviation of the six algorithms in planning time, number of path nodes, and path length show a decreasing trend, with the improved RRT* algorithm exhibiting the smallest variance and standard deviation. This indicates that the improved RRT* algorithm exhibits significantly greater stability in these metrics, reflecting minimal fluctuation and highlighting the superiority of the enhanced algorithm.

### 5.2. Simulation and Analysis in a Complex Environment

**Figure 12:**
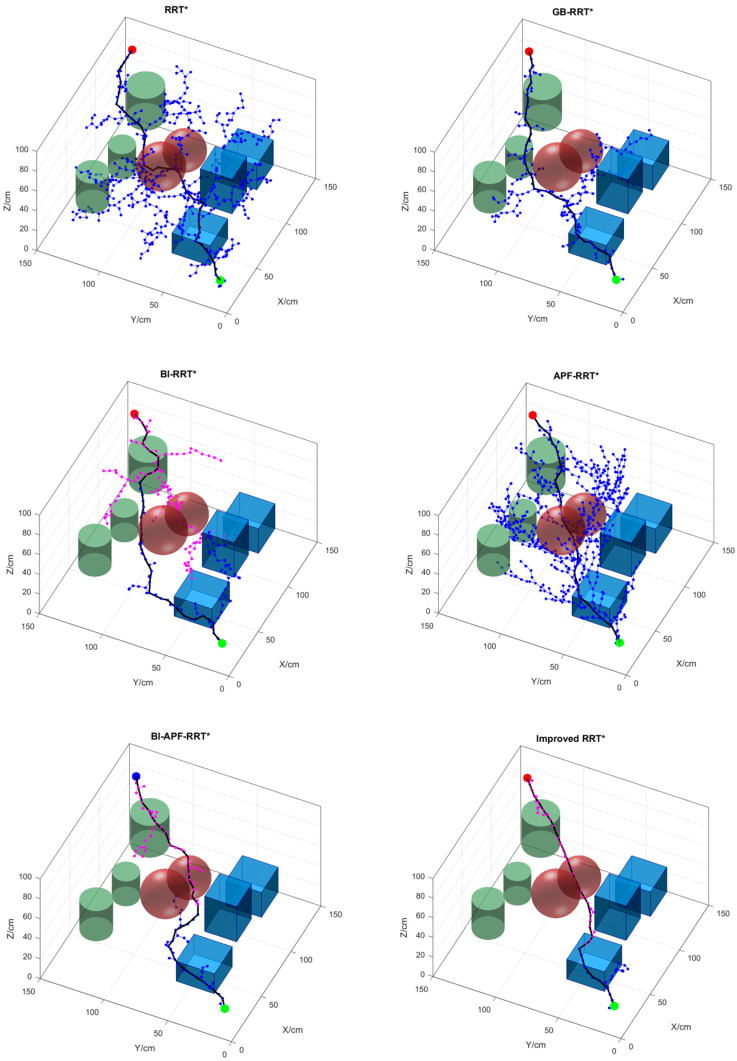
Obstacle-avoidance path planning results of the six algorithms in a complex environment. The green sphere represents the starting point (10 cm, 10 cm, 20 cm), the red sphere represents the goal point (140 cm, 140 cm, 80 cm), the light red and purple points represent the sampling points, the cube, cuboid, sphere, and cylinder represent obstacles, and the solid lines represent the final paths planned by each algorithm.

Figure 12 shows the obstacle-avoidance path planning results of six different algorithms in a complex environment, with their comparative performance summarized in Table 3. As can be seen from Figure 12, the advantages of the proposed algorithm become even more pronounced in this complex setting. Although unidirectional search methods have certain merits, bidirectional search achieves shorter planning times—yet not necessarily better paths. The algorithm proposed in this paper implements bidirectional search, significantly reducing both planning time and a large number of redundant nodes.

By analyzing the data in Table 6, in the complex map environment, the improved RRT* algorithm reduces planning time by 88.60%, 66.67%, 61.76%, 87.74%, and 76.79% compared to RRT*, GB-RRT*, BI-RRT*, APF-RRT*, and BI-APF-RRT*, respectively. The number of path nodes is reduced by 90.09%, 72.73%, 31.82%, 88.82%, and 53.42%, respectively. The path length is shortened by 20.50%, 13.11%, 8.63%, 10.39%, and 14.45%, respectively. The improved RRT* algorithm proposed in this paper demonstrates significant advantages in 3D environments: planning time is markedly reduced, the number of redundant nodes is substantially decreased, and the resulting obstacle-avoidance paths are notably smoother.

To better analyze the stability of the six algorithms, 30 experiments were conducted. Based on data analysis, the variance and standard deviation of the planning time, number of path nodes, and path length for the six algorithms across the 30 experiments are shown in Table 7, Table 8 and Table 9, respectively.

Based on the data analysis from Table 7, Table 8 and Table 9, the six algorithms demonstrate varying performance in planning time, number of path nodes, and path length within a complex map environment. Specifically, the variance and standard deviation of the six algorithms in planning time, number of path nodes, and path length show a decreasing trend, with the improved RRT* algorithm exhibiting the smallest variance and standard deviation. This indicates that the improved RRT* algorithm exhibits significantly greater stability in these metrics, reflecting minimal fluctuation and highlighting the superiority of the enhanced algorithm.

**Figure 13:**
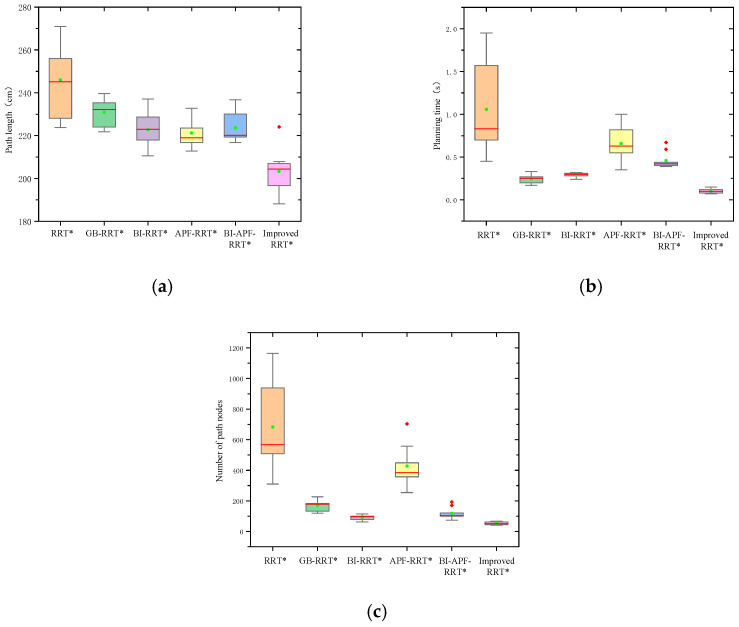
Algorithm performance comparison in a simple environment. (**a**) Path length comparison; (**b**) planning time comparison; (**c**) comparison of the number of path nodes. The red line in the chart represents the median, the green dots represent the average values, and the red diamonds represent outliers.

**Figure 14:**
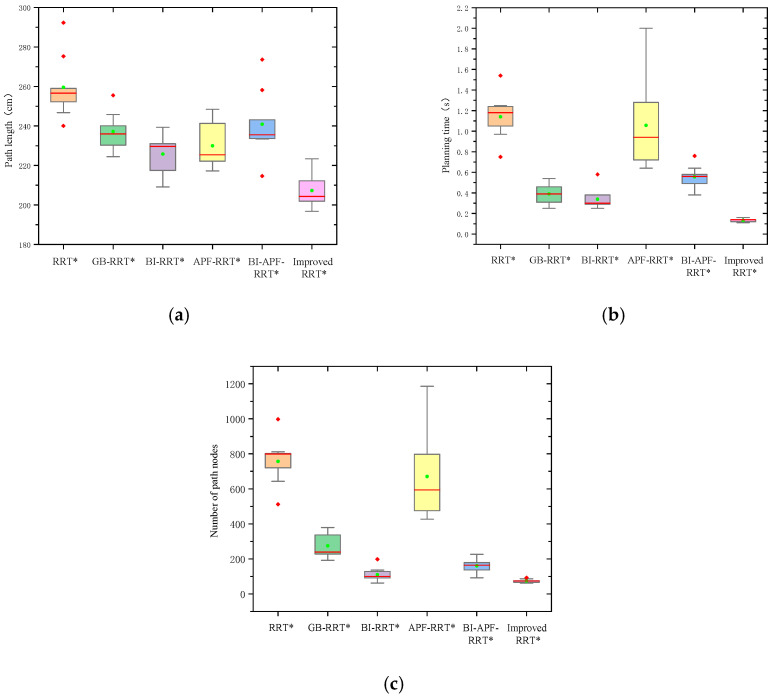
Algorithm performance comparison in a complex environment. (**a**) Path length comparison; (**b**) planning time comparison; (**c**) comparison of the number of path nodes. The red line in the chart represents the median, the green dots represent the average values, and the red diamonds represent outliers.

As shown in Figure 13 and Figure 14, the path length, planning time, and number of nodes of the trajectories generated by all algorithms in both environments are compared. Each algorithm was executed 30 times, and box plots of path lengths are presented in Figure 13a and Figure 14a. In both environments, the paths generated by the proposed algorithm are consistently shorter than those produced by the other algorithms. Figure 13b and Figure 14b display the comparison of planning time across the six algorithms. Clearly, the proposed algorithm requires less time than the others, with its advantage being particularly pronounced in the complex environment. The box plots of node counts for all algorithms are shown in Figure 13c and Figure 14c. The improved RRT* algorithm yields the fewest nodes in both environments, indicating superior tracking performance compared to the other algorithms. 

### 5.3. Statistical Significance Analysis

To quantitatively assess the statistical significance of differences in performance metrics between the improved RRT* algorithm and other comparative algorithms, this paper adopts the Friedman test combined with the Nemenyi post hoc test for analysis. This non-parametric testing method is suitable for the repeated measures design of this experiment (30 independent runs) and does not require the data to follow a normal distribution.

Separate tests were conducted on three core metrics in both simple and complex environments—planning time, number of path nodes, and path length. The testing procedure was as follows:

(1) Data preparation: Organize the corresponding metric data from the six algorithms (RRT*, GB-RRT*, BI-RRT*, APF-RRT*, BI-APF-RRT*, Improved RRT*) in the 30 experiments;
(2) Intra-group ranking: For each experiment, the six results are ranked (best as 1, worst as 6), and calculations are performed;
(3) Friedman test: Calculate the test statistic using the formula $\chi_{F}^{2}=\frac{12N}{k^{\prime }(k^{\prime }+1)}\left[\sum_{j=1}^{k^{\prime }}R_{j}^{2}-\frac{k^{\prime }{\left(k^{\prime }+1\right)}^{2}}{4}\right]$, where N=30, and $k^{\prime }=6$;
(4) Significance determination: Determine the p-value based on the chi-square distribu-tion with degrees of freedom $d_{f}=k^{\prime }-1=5$;
(5) Post hoc comparison: If p<0.05, then proceed with the Nemenyi test for pairwise comparisons using $CD=q_{\alpha_{3},k^{\prime },\infty }\sqrt{\frac{k^{\prime }(k^{\prime }+1)}{6N}}=q_{0.05,6,\infty }\sqrt{\frac{6\times 7}{6\times 30}}$.

#### 5.3.1. Friedman Test Analysis in Simple Environments

Performance comparison of planning time, number of path nodes, and path length among six algorithms in the test analysis is shown in the table below. The null hypothesis $H_{0}$ is that “the path lengths of all algorithms are equal”, and a rank difference greater than 1.418 indicates significance at the $\alpha_{3}=0.05$ level.

As shown in Table 10, Table 11, Table 12 and Table 13, the Friedman test indicates that each algorithm exhibits varying performance in terms of planning time, number of path nodes, and path length. Among them, the improved RRT* algorithm demonstrates statistically significant superiority over the other algorithms, consistently achieving the highest average ranking. The p-value ranges also fall within acceptable limits, further confirming the stability of the improved RRT* algorithm and highlighting its overall superiority.

#### 5.3.2. Friedman Test Analysis in Complex Environments

To verify the performance comparison of planning time, number of path nodes, and path length among the six algorithms in complex environments, a Friedman test analysis was conducted on the test indicators, with the results presented in the table below. The null hypothesis $H_{0}$ is that “the path lengths of all algorithms are equal”, and a rank difference greater than 1.418 indicates significance at the $\alpha_{3}=0.05$ level.

As shown in Table 14, Table 15, Table 16 and Table 17, the Friedman test indicates that each algorithm exhibits varying performance in terms of planning time, number of path nodes, and path length. Among them, the improved RRT* algorithm demonstrates statistically significant superiority over the other algorithms, consistently achieving the highest average ranking. The p-value ranges also fall within acceptable limits, further confirming the stability of the improved RRT* algorithm and highlighting its overall superiority.

## 6. Physical Robot Experiment

**Figure 15:**
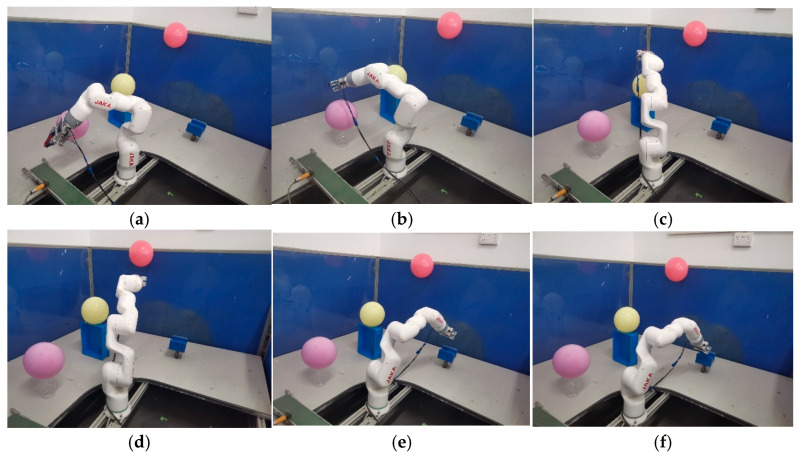
Robotic arm motion process. (**a**) Initial position; (**b**) waypoint 1; (**c**) waypoint 2; (**d**) waypoint 3; (**e**) waypoint 4; (**f**) goal point.

To evaluate the effectiveness of the proposed algorithm in real-world applications, physical experiments were conducted using a JAKA robotic arm. In the experiment, a balloon was used to simulate an obstacle, and a square box served as the target location, establishing a test environment consistent with real-world operating conditions. The obstacle-avoidance path planning algorithm was executed on a host computer, which generated motion commands and transmitted them to the robot controller via the JAKA robot API, thereby driving the robotic arm to complete the obstacle-avoidance task. Figure 15 illustrates the physical experimental setup. As shown, the robotic arm successfully avoided the obstacle and accurately reached the target position, demonstrating that the proposed algorithm is both feasible and practical in real-world scenarios.

**Figure 16:**
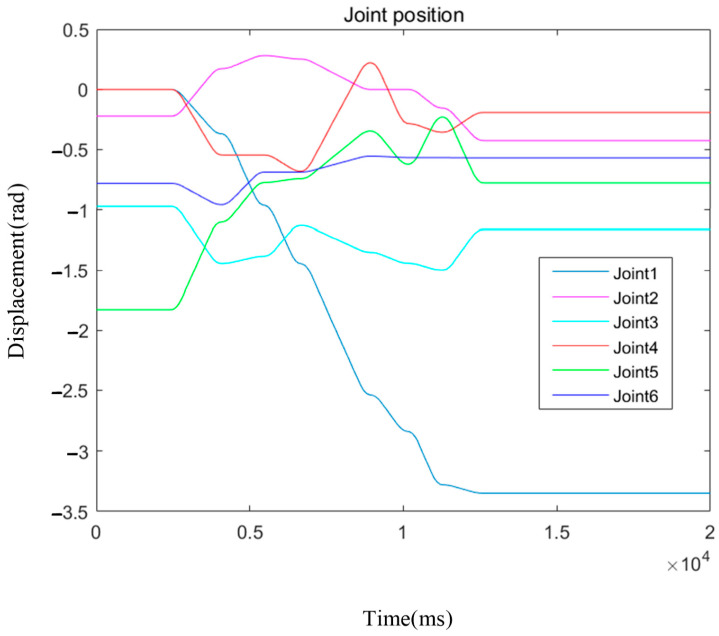
Position of each joint.

Furthermore, to gain deeper insight into the performance of each joint of the robotic arm during motion, a plot of joint positions over time was generated (see Figure 16). This figure clearly illustrates the motion trajectories of all six joints during path execution. It can be observed that joints 1, 4, and 5 exhibit more pronounced position changes, indicating their critical roles in overall motion coordination. Specifically, joints 4 and 5 are primarily responsible for adjusting the posture of the middle section of the arm, ensuring smooth transitions along the planned path. In contrast, joint 6 mainly focuses on fine-tuning the orientation of the end-effector. The smoothness of these trajectory curves further demonstrates the stability and good controllability of the improved RRT* algorithm in guiding robotic arm motion.

The experiment was repeated 30 times, and the statistical results—including average planning time, average path length, and the success rate of the robotic arm in completing the task—are summarized in Table 18.

Experimental results demonstrate that the improved RRT* algorithm significantly outperforms all other algorithms across all performance metrics. Compared with RRT*, GB-RRT*, BI-RRT*, APF-RRT*, and BI-APF-RRT*, the improved RRT* algorithm reduces planning time by 58.46%, 46.75%, 36.49%, 39.78%, and 22.72%, respectively; shortens path length by 18.25%, 13.32%, 10.04%, 8.98%, and 5.21%, respectively; and increases the robotic arm’s task success rate by approximately 21.8%—rising from 78.2% (with the standard RRT*) to 100%. The reduction in planning time indicates that the improved RRT* algorithm optimizes both the sampling mechanism and node selection efficiency during path search. The shorter path length demonstrates its ability to generate higher-quality motion trajectories, thereby reducing motion redundancy. Moreover, the improved success rate further validates the enhanced robustness and reliability of the proposed algorithm, particularly in complex and obstacle-dense environments. Collectively, these experimental results confirm that the improved RRT* algorithm can efficiently plan paths, effectively avoid obstacles, and reliably guide the robotic arm to reach its target position safely and accurately.

## 7. Conclusions

To address several limitations of the standard RRT* algorithm when applied to robotic arm path planning—such as insufficient goal bias in random sampling, slow convergence, low path search efficiency, and lack of path smoothness—this paper presents a comprehensive study and proposes an improved RRT* algorithm. First, a dynamic ellipsoidal sampling strategy is introduced to adaptively shrink the sampling region, accelerating spatial exploration. The size of the ellipsoid is dynamically adjusted based on search progress and environmental complexity, thereby enhancing the quality of sampled points. Second, a bidirectional RRT* framework is adopted, where trees grow alternately from both the start and goal points, significantly improving convergence efficiency. Additionally, a dynamic goal-biased strategy is employed to greedily steer the random tree toward the goal, enhancing planning speed. A heuristic search mechanism is integrated with RRT* to dynamically balance heuristic guidance and random exploration, further accelerating convergence. Moreover, an enhanced artificial potential field (APF) method is used to guide tree expansion, effectively reducing the number of iterations required. To optimize local path quality, a local reconnection optimization strategy is applied to lower the accumulated cost to come of newly added nodes while simultaneously balancing path length, smoothness, and safety. Finally, path pruning removes redundant nodes, and cubic B-spline interpolation is used to smooth the final trajectory. During smoothing, the cubic B-spline algorithm refines the path to achieve optimal continuity and smoothness. Three-dimensional simulation results demonstrate that, compared with RRT*, GB-RRT* (goal-biased RRT*), BI-RRT* (bidirectional RRT*), APF-RRT*, and BI-APF-RRT*, the proposed improved RRT* algorithm exhibits significant advantages in both planning efficiency and path quality. Specifically, it achieves shorter planning times, fewer path nodes, and reduced path lengths while generating trajectories with superior smoothness and continuity—substantially enhancing the robotic arm’s path planning performance in 3D environments.

## Figures and Tables

**Table 1:** Robotic arm D-H parameters.

Joint i	$\theta_{i}/rad$	$d_{i}/mm$	$a_{i}/mm$	$\alpha_{i}/rad$
Joint 1	$\theta_{1}$	187	0	−90
Joint 2	$\theta_{2}$	6	210	0
Joint 3	$\theta_{3}$	0	0	90
Joint 4	$\theta_{4}$	210	0	−90
Joint 5	$\theta_{5}$	0	0	90
Joint 6	$\theta_{6}$	159	0	0

**Table 2:** Comparison of algorithms in a simple environment.

Algorithm	Average Planning Time/s	Average Number of Path Nodes	Average Path Length/cm	Average Path Safety Distance/cm
RRT*	1.06	684.00	246.02	2.41
GB-RRT*	0.24	171.00	230.87	2.66
BI-RRT*	0.29	92.00	222.76	2.79
APF-RRT*	0.66	429.00	221.20	3.52
BI-APF-RRT*	0.46	118.00	223.67	2.55
Improved RRT*	0.10	55.00	203.30	3.50

**Table 3:** Comparison of variance and standard deviation in planning time for each algorithm in a simple environment.

Algorithm	Variance	Standard Deviation
RRT*	0.174	0.417
GB-RRT*	0.0095	0.097
BI-RRT*	0.0036	0.060
APF-RRT*	0.014	0.118
BI-APF-RRT*	0.0041	0.064
Improved RRT*	0.00025	0.016

**Table 4:** Comparison of variance and standard deviation in the number of path nodes for each algorithm in a simple environment.

Algorithm	Variance	Standard Deviation
RRT*	55,842.22	236.31
GB-RRT*	1056.21	32.50
BI-RRT*	220.33	14.84
APF-RRT*	638.67	25.27
BI-APF-RRT*	117.33	10.83
Improved RRT*	18.99	4.36

**Table 5:** Comparison of variance and standard deviation in path length for each algorithm in a simple environment.

Algorithm	Variance	Standard Deviation
RRT*	320.42	17.90
GB-RRT*	56.87	7.54
BI-RRT*	90.15	9.49
APF-RRT*	44.76	6.69
BI-APF-RRT*	45.12	6.72
Improved RRT*	12.34	3.51

**Table 6:** Comparison of algorithms in a complex environment.

Algorithm	Average Planning Time/s	Average Number of Path Nodes	Average Path Length/cm	Average Path Safety Distance/cm
RRT*	1.14	757.00	259.45	2.87
GB-RRT*	0.39	275.00	237.40	2.56
BI-RRT*	0.34	110.00	225.76	2.66
APF-RRT*	1.06	671.00	230.19	3.06
BI-APF-RRT*	0.56	161.00	241.11	3.34
Improved RRT*	0.13	75.00	206.27	3.69

**Table 7:** Comparison of variance and standard deviation in planning time for each algorithm in a complex environment.

Algorithm	Variance	Standard Deviation
RRT*	0.074	0.272
GB-RRT*	0.032	0.179
BI-RRT*	0.014	0.118
APF-RRT*	0.0064	0.080
BI-APF-RRT*	0.0025	0.050
Improved RRT*	0.00021	0.014

**Table 8:** Comparison of variance and standard deviation in the number of path nodes for each algorithm in a complex environment.

Algorithm	Variance	Standard Deviation
RRT*	38,700	196.7
GB-RRT*	5600	110.9
BI-RRT*	3100	55.7
APF-RRT*	15,800	125.7
BI-APF-RRT*	900	30.0
Improved RRT*	70	8.4

**Table 9:** Comparison of variance and standard deviation in path length for each algorithm in a complex environment.

Algorithm	Variance	Standard Deviation
RRT*	349.8	18.70
GB-RRT*	233.1	15.27
BI-RRT*	156.3	12.50
APF-RRT*	107.5	10.37
BI-APF-RRT*	64.8	8.05
Improved RRT*	39.6	6.29

**Table 10:** Results of the Friedman test in simple environments.

Test Indicators	Test Items	Statistic	Degrees of Freedom	p-Value	Significance Level	Conclusion
planning time	Friedman test	*χ*^2^ = 114.5	5	<0.00001	$\alpha_{3}=0.05$	Reject the null hypothesis
	Nemenyi critical difference	CD=1.418	-	-	-	Pairwise comparison benchmark
number of path nodes	Friedman test	*χ*^2^ = 302.1	5	<0.00001	$\alpha_{3}=0.05$	Reject the null hypothesis
	Nemenyi critical difference	CD=1.418	-	-	-	Pairwise comparison benchmark
path length	Friedman test	*χ*^2^ = 182.9	5	<0.00001	$\alpha_{3}=0.05$	Reject the null hypothesis
	Nemenyi critical difference	CD=1.418	-	-	-	Pairwise comparison benchmark

**Table 11:** Average ranking of planning time for each algorithm and significance of pairwise comparisons.

Algorithm	$\mathrm{Average}\;\mathrm{Rank}\;R_{j}$	Rank Difference from Improved RRT*	Statistical Significance (CD=1.418)	p-Value Range
Improved RRT*	1.00	-	-	-
BI-APF-RRT*	2.70	1.70	Significant	p < 0.01
BI-RRT*	3.20	2.20	Significant	p < 0.001
GB-RRT*	3.80	2.80	Significant	p < 0.001
APF-RRT*	4.50	3.50	Significant	p < 0.001
RRT*	5.80	4.80	Significant	p < 0.001

**Table 12:** Average ranking of path node count for each algorithm and significance of pairwise comparisons.

Algorithm	$\mathrm{Average}\;\mathrm{Rank}\;R_{j}$	Rank Difference from Improved RRT*	Statistical Significance (CD=1.418)	p-Value Range
Improved RRT*	1.00	-	-	-
BI-RRT*	3.00	2.00	Significant	p < 0.01
BI-APF-RRT*	3.50	2.50	Significant	p < 0.001
GB-RRT*	4.50	3.50	Significant	p < 0.001
APF-RRT*	5.50	4.50	Significant	*p* < 0.001
RRT*	6.00	5.00	Significant	p < 0.001

**Table 13:** Average ranking of path length for each algorithm and significance of pairwise comparisons.

Algorithm	$\mathrm{Average}\;\mathrm{Rank}\;R_{j}$	Rank Difference from Improved RRT*	Statistical Significance (CD=1.418)	p-Value Range
Improved RRT*	1.00	-	-	-
BI-APF-RRT*	2.70	1.70	Significant	*p* < 0.01
APF-RRT*	3.60	2.60	Significant	*p* < 0.001
BI-RRT*	4.00	3.00	Significant	*p* < 0.001
GB-RRT*	4.93	3.93	Significant	*p* < 0.001
RRT*	5.77	4.77	Significant	*p* < 0.001

**Table 14:** Results of the Friedman test in complex environments.

Test Indicators	Test Items	Statistic	Degrees of Freedom	p-Value	Significance Level	Conclusion
planning time	Friedman test	*χ*^2^ = 192.3	5	<0.00001	$\alpha_{3}=0.05$	Reject the null hypothesis
	Nemenyi critical difference	CD=1.418	-	-	-	Pairwise comparison benchmark
number of path nodes	Friedman test	*χ*^2^ = 125.4	5	<0.00001	$\alpha_{3}=0.05$	Reject the null hypothesis
	Nemenyi critical difference	CD=1.418	-	-	-	Pairwise comparison benchmark
path length	Friedman test	*χ*^2^ = 375.0	5	<0.00001	$\alpha_{3}=0.05$	Reject the null hypothesis
	Nemenyi critical difference	CD=1.418	-	-	-	Pairwise comparison benchmark

**Table 15:** Average ranking of planning time for each algorithm and significance of pairwise comparisons.

Algorithm	$\mathrm{Average}\;\mathrm{Rank}\;R_{j}$	Rank Difference from Improved RRT*	Statistical Significance (CD=1.418)	p-Value Range
Improved RRT*	1.00	-	-	-
BI-RRT*	2.50	1.50	Significant	*p* < 0.001
GB-RRT*	3.40	2.40	Significant	*p* < 0.001
BI-APF-RRT*	4.30	3.30	Significant	*p* < 0.001
APF-RRT*	5.00	4.00	Significant	*p* < 0.001
RRT*	5.80	4.80	Significant	*p* < 0.001

**Table 16:** Average ranking of path node count for each algorithm and significance of pairwise comparisons.

Algorithm	$\mathrm{Average}\;\mathrm{Rank}\;R_{j}$	Rank Difference from Improved RRT*	Statistical Significance (CD=1.418)	p-Value Range
Improved RRT*	1.00	-	-	-
BI-RRT*	2.67	1.67	Significant	*p* < 0.01
BI-APF-RRT*	2.83	1.83	Significant	*p* < 0.001
GB-RRT*	3.83	2.83	Significant	*p* < 0.001
APF-RRT*	4.83	3.83	Significant	*p* < 0.001
RRT*	5.83	4.83	Significant	*p* < 0.001

**Table 17:** Average ranking of path length for each algorithm and significance of pairwise comparisons.

Algorithm	$\mathrm{Average}\;\mathrm{Rank}\;R_{j}$	Rank Difference from Improved RRT*	Statistical Significance (CD=1.418)	p-Value Range
Improved RRT*	1.00	-	-	-
BI-APF-RRT*	3.00	2.00	Significant	*p* < 0.01
APF-RRT*	4.00	3.00	Significant	*p* < 0.001
BI-RRT*	5.00	4.00	Significant	*p* < 0.001
GB-RRT*	5.50	4.50	Significant	*p* < 0.001
RRT*	6.00	5.00	Significant	*p* < 0.001

**Table 18:** Robotic arm motion planning results.

Algorithm	Average Planning Time/s	Average Path Length/cm	Success Rate of Robotic Arm Movement/Percent
RRT*	5.32	75.6	78.2
GB-RRT*	4.15	71.3	84.5
BI-RRT*	3.48	68.7	88.1
APF-RRT*	3.67	67.9	90.3
BI-APF-RRT*	2.86	65.2	93.8
Improved RRT*	2.21	61.8	100

## Data Availability

The data in this study are available upon request from the corresponding author.

## References

[1] Zeng A., Yu K.-T., Song S., Suo D., Walker E., Rodriguez A., Xiao J. Multi-View Self-Supervised Deep Learning for 6D Pose Estimation in the Amazon Picking Challenge. Proceedings of the 2017 IEEE International Conference on Robotics and Auto-mation (ICRA).

[2] Suarez A., Heredia G., Ollero A. (2018). Physical-virtual impedance control in ultralightweight and compliant dual-arm aerial manipulators. IEEE Rob. Autom. Lett..

[3] Wang X., Ma X., Li X., Ma X., Li C. (2022). Target-biased informed trees: Sampling-based method for optimal motion planning in complex environments. J. Comput. Des. Eng..

[4] Wang X., Yang Y., Wang D., Zhang Z. (2022). Mission-oriented cooperative 3D path planning for modular solar-powered aircraft with energy optimization. Chin. J. Aeronaut..

[5] Alshammrei S., Boubaker S., Kolsi L. (2022). Improved Dijkstra algorithm for mobile robot path planning and obstacle avoidance. Comput. Mater. Contin..

[6] Erke S., Bin D., Yiming N., Qi Z., Liang X., Dawei Z. (2020). An improved A-Star based path planning algorithm for autonomous land vehicles. Int. J. Adv. Robot. Syst..

[7] Jin J., Zhang Y., Zhou Z., Jin M., Yang X., Hu F. (2023). Conflict-based search with D* lite algorithm for robot path planning in unknown dynamic environments. Comput. Electr. Eng..

[8] Taheri E., Ferdowsi M.H., Danesh M. (2018). Fuzzy greedy RRT path planning algorithm in a complex configuration space. Int. J. Control Autom..

[9] Qi J., Yang H., Sun H. (2020). MOD-RRT*: A sampling-based algorithm for robot path planning in dynamic environment. IEEE Trans. Ind. Electron..

[10] Qureshi A.H., Ayaz Y. (2016). Potential functions based sampling heuristic for optimal path planning. Auton. Robot..

[11] Guo S., Gong J., Shen H., Yuan L., Wei W., Long Y. (2025). DBVSB-P-RRT*: A path planning algorithm for mobile robot with high environmental adaptability and ultra-high speed planning. Expert Syst. Appl..

[12] Jiang Z., Liu Q., Wang E. (2025). HS-APF-RRT*: An Off-Road Path-Planning Algorithm for Unmanned Ground Vehicles Based on Hierarchical Sampling and an Enhanced Artificial Potential Field. Comput. Mater. Contin..

[13] Jeong I.B., Lee S.J., Kim J.H. (2019). Quick-RRT*: Triangular inequality-based implementation of RRT* with improved initial solution and convergence rate. Expert Syst. Appl..

[14] Li Y., Wei W., Gao Y., Wang D., Fan Z. (2020). PQ-RRT*: An improved path planning algorithm for mobile robots. Expert Syst. Appl..

[15] Wei K., Ren B. (2018). A method on dynamic path planning for robotic manipulator autonomous obstacle avoidance based on an improved RRT algorithm. Sensors.

[16] Liao B., Wan F., Hua Y., Ma R., Zhu S., Qing X. (2021). F-RRT*: An improved path planning algorithm with improved initial solution and convergence rate. Expert Syst. Appl..

[17] Cong J., Hu J., Wang Y., He Z., Han L., Su M. (2023). FF-RRT*: A sampling-improved path planning algorithm for mobile robots against concave cavity obstacle. Complex. Intell. Syst..

[18] Chai Q., Wang Y. (2022). RJ-RRT: Improved RRT for path planning in narrow passages. Appl. Sci..

[19] Ganesan S., Ramalingam B., Mohan R.E. (2024). A hybrid sampling-based RRT* path planning algorithm for autonomous mobile robot navigation. Expert Syst. Appl..

[20] Qie T., Wang W., Yang C., Li Y., Liu W., Xiang C. (2022). A path planning algorithm for autonomous flying vehicles in cross-country environments with a novel TF-RRT∗ method. Green Energy Intell..

[21] Ji H., Xie H., Wang C., Yang H. (2023). E-RRT*: Path planning for hyper-redundant manipulators. IEEE Rob. Autom. Lett..

[22] Ding J., Zhou Y., Huang X., Song K., Lu S., Wang L. (2023). An improved RRT* algorithm for robot path planning based on path expansion heuristic sampling. J. Comput. Sci..

[23] Chen L., Shan Y., Tian W., Li B., Cao D. (2018). A fast and efficient double-tree RRT*-like sampling-based planner applying on mobile robotic systems. IEEE/ASME Trans. Mechatron..

[24] Chao N., Liu Y.K., Xia H., Ayodeji A., Bai L. (2018). Grid-based RRT* for minimum dose walking path-planning in complex radioactive environments. Ann. Nucl. Energy.

[25] Cheng X., Zhou J., Zhou Z., Zhao X., Gao J., Qiao T. (2023). An improved RRT-Connect path planning algorithm of robotic arm for automatic sampling of exhaust emission detection in Industry 4.0. J. Ind. Inf. Integr..

[26] Huang C., Tang B., Guo Z., Su Q., Gai J. (2024). Agile-rrt*: A faster and more robust path planner with enhanced initial solution and convergence rate in complex environments. IEEE Access.

